# A Mobile Health App for the Collection of Functional Outcomes After Inpatient Stroke Rehabilitation: Pilot Randomized Controlled Trial

**DOI:** 10.2196/17219

**Published:** 2020-05-13

**Authors:** Li Li, Jia Huang, Jingsong Wu, Cai Jiang, Shanjia Chen, Guanli Xie, Jinxin Ren, Jing Tao, Chetwyn C H Chan, Lidian Chen, Alex W K Wong

**Affiliations:** 1 College of Rehabilitation Medicine Fujian University of Traditional Chinese Medicine Fuzhou China; 2 Key Laboratory of Orthopedics & Traumatology of Traditional Chinese Medicine and Rehabilitation Fujian University of Traditional Chinese Medicine, Ministry of Education Fuzhou China; 3 Traditional Chinese Medicine Rehabilitation Research Center of State Administration of Traditional Chinese Medicine Fuzhou China; 4 Department of Rehabilitation Sciences The Hong Kong Polytechnic University HongKong Hong Kong; 5 Program in Occupational Therapy Washington University School of Medicine St. Louis, MO United States; 6 Department of Neurology Washington University School of Medicine St. Louis, MO United States; 7 Department of Psychiatry Washington University School of Medicine St. Louis, MO United States

**Keywords:** telemedicine, cell phone, stroke, rehabilitation, activities of daily living, outcome and process assessment, health care

## Abstract

**Background:**

Monitoring the functional status of poststroke patients after they transition home is significant for rehabilitation. Mobile health (mHealth) technologies may provide an opportunity to reach and follow patients post discharge. However, the feasibility and validity of functional assessments administered by mHealth technologies are unknown.

**Objective:**

This study aimed to evaluate the feasibility, validity, and reliability of functional assessments administered through the videoconference function of a mobile phone–based app compared with administration through the telephone function in poststroke patients after rehabilitation hospitalization.

**Methods:**

A randomized controlled trial was conducted in a rehabilitation hospital in Southeast China. Participants were randomly assigned to either a videoconference follow-up (n=60) or a telephone follow-up (n=60) group. We measured the functional status of participants in each group at 2-week and 3-month follow-up periods. Half the participants in each group were followed by face-to-face home visit assessments as the gold standard. Validity was assessed by comparing any score differences between videoconference follow-up and home visit assessments, as well as telephone follow-up and home visit assessments. Reliability was assessed by computing agreements between videoconference follow-up and home visit assessments, as well as telephone follow-up and home visit assessments. Feasibility was evaluated by the levels of completion, satisfaction, comfort, and confidence in the 2 groups.

**Results:**

Scores obtained from the videoconference follow-up were similar to those of the home visit assessment. However, most scores collected from telephone administration were higher than those of the home visit assessment. The agreement between videoconference follow-up and home visit assessments was higher than that between telephone follow-up and home visit assessments at all follow-up periods. In the telephone follow-up group, completion rates were 95% and 82% at 2-week and 3-month follow-up points, respectively. In the videoconference follow-up group, completion rates were 95% and 80% at 2-week and 3-month follow-up points, respectively. There were no differences in the completion rates between the 2 groups at all follow-up periods (*X*^2^_1_=1.6, *P*=.21 for 2-week follow-up; *X*^2^_1_=1.9, *P*=.17 for 3-month follow-up). Patients in the videoconference follow-up group perceived higher confidence than those in the telephone follow-up group at both 2-week and 3-month follow-up periods (*X*^2^_3_=6.7, *P*=.04 for 2-week follow-up; *X*^2^_3_=8.0, *P*=.04 for 3-month follow-up). The videoconference follow-up group demonstrated higher satisfaction than the telephone follow-up group at 3-month follow-up (*X*^2^_3_=13.9; *P*=.03).

**Conclusions:**

The videoconference follow-up assessment of functional status demonstrates higher validity and reliability, as well as higher confidence and satisfaction perceived by patients, than the telephone assessment. The videoconference assessment provides an efficient means of assessing functional outcomes of patients after hospital discharge. This method provides a novel solution for clinical trials requiring longitudinal assessments.

**Trial Registration:**

chictr.org.cn: ChiCTR1900027626; http://www.chictr.org.cn/edit.aspx?pid=44831&htm=4.

## Introduction

Stroke is one of the leading causes of death and disability worldwide [1]. As in other developing countries, the incidence and prevalence of stroke in China are gradually increasing. Each year, China has 2.5 million new stroke cases and more than 11 million stroke survivors; stroke has become the leading cause of death in China [2]. Stroke can have a long-term impact on an individual’s physical, mental, and social function, as well as on survivors’ caregivers and families [3-6]. The majority of patients receive inpatient stroke rehabilitation to regain function for only the first few weeks after stroke, but functional recovery often occurs 3 months or even longer following a stroke [7]. Additionally, about 30% of poststroke individuals receive outpatient rehabilitation [8]. Even if available, access to rehabilitation for patients in China is limited because of transportation, geographical barriers, and monetary factors [9]. As most patients have recovery potential but do not receive recommended rehabilitation, it is important to develop new strategies to continuously monitor functional recovery and other health outcomes of patients following discharge to better understand the long-term consequences for patients poststroke [10].

Poststroke home-based therapies seem to be a viable option for the delivery of stroke care. Follow-up assessments and interventions not only provide a means of monitoring the functional status of patients after transitioning to home and community [11], but also provide instructions to prevent readmission, which is especially important for those who receive a longer stay in inpatient rehabilitation [12]. Moreover, follow-up assessments enable clinicians to adjust the treatment plan for home-based therapies [13,14]. One common method for follow-up assessment is a face-to-face, at-home assessment in which the home health therapist visits the patient at home to perform an evaluation. However, this method demands intensive resources, including the time of trained personnel and financial expenditures [15]. Recent studies have tested alternative methods of follow-up data collection for patients following a stroke [16-18].

Telephone administration is a common alternative. This method allows participants to be recruited from diverse geographical areas, is typically less expensive than the face-to-face home assessment, and has a quick turnaround time [19]. Prior studies have found that telephone administration of outcome measures demonstrates equivalent reliability to face-to-face assessment, supporting telephone interview as a feasible solution [20,21]. However, there are some shortcomings to telephone administration. First, many functional measures require a trained therapist to observe and provide ratings of how the patient performs specific daily tasks. Inability to perform observations via telephone administration may be a hindrance to accurately evaluating task performance. Furthermore, telephone administration often assesses survey-based questions, which require patients to have higher education, health literacy, and communication abilities to understand the verbal instructions [22]. An earlier study found a large amount of missing data from assessments administered through the telephone interview method for stroke patients and caregivers, limiting the use of this method in clinical trials requiring longitudinal assessments [23].

With advances in computing power and mobile connectivity, many mobile health (mHealth) technologies, such as mobile devices, sensors, apps, and social media, are becoming available to obtain data pertinent to wellness and disease diagnosis, prevention, and management [24]. WeChat (Chinese version: Weixin), developed in 2011 by Tencent, has become the most common social software app in China [25]. Similar to other social media apps, such as Facebook, Twitter, and WhatsApp, WeChat is a free platform that provides seamless opportunities for communication and other mobile apps. People can communicate with one another through the free voice call or video call feature, as well as instantly share information [26]. According to the Statista Research Department [27], WeChat had over 1.15 billion monthly active users from a wide range of age groups. Harnessing the use of mHealth technologies to improve health and wellness is not uncommon in modern health care [28]. A recent mHealth intervention study utilizing the WeChat app for weight loss behaviors in a group of male workers found promising results: participants who spent more time using the health education program embedded in the WeChat app for engaging in healthy behaviors demonstrated more weight loss [29]. Another mHealth study used the WeChat app to educate parents of pediatric patients undergoing surgery and found that this mobile app–assisted intervention was effective in enhancing parents’ knowledge of perioperative procedures [30]. Another study used the WeChat app in a group of discharged patients with head and neck tumors for 6 months and demonstrated the app to be a cost-effective method of follow-up assessment [31].

Although the utility of the WeChat app has been demonstrated in other populations, little is known about whether the WeChat app could be feasible to assess the postdischarge functional status of patients following a stroke, because most poststroke patients experience cognitive and communication difficulties that may make it difficult to operate the app and understand its instructions. To address this question, we conducted a pilot randomized controlled trial in a group of discharged stroke patients by randomly assigning them into 2 different modes of administration during 2-week and 3-month follow-up periods: WeChat video conference or WeChat telephone administration. This study had 2 specific aims. The first aim was to compare the validity and reliability of functional assessment between these 2 modes of administration in stroke patients. We hypothesized that videoconference administration would demonstrate higher validity and reliability than telephone administration, because examiners using videoconference administration can observe how the respondent performs specific tasks to provide appropriate ratings, whereas examiners in the telephone administration group demand more subjective appraisals of task performance based on the respondent’s verbal descriptions. Our second aim was to examine the feasibility of the functional assessment administered via the videoconference function compared with the telephone call function in stroke patients after rehabilitation hospitalization. We hypothesized that both modes of administration would demonstrate high levels of completion, comfort, satisfaction, and confidence.

## Methods

### Study Design

This study was a parallel, 2-group, and pragmatic randomized controlled trial of an mHealth app of functional outcome data collection after inpatient stroke rehabilitation. The trial was registered with the Chinese Clinical Trial Registry: ChiCTR1900027626. A total of 120 eligible stroke patients from the affiliated rehabilitation hospital of Fujian University of Traditional Chinese Medicine were recruited for this study. Participants were approached by a research assistant, who provided the study information. After participants provided informed consent and were screened for eligibility, participants were randomized into 1 of the 2 WeChat app administration groups: videoconference follow-up or telephone follow-up, with a ratio of 1:1. Eligible participants were randomized using a random number table generated by a study coordinator who was not involved in the recruitment and assessment of participants for the study.

### Ethics Approval

This trial was implemented in compliance with the declaration of Helsinki and approved by the Ethics Board of the affiliated rehabilitation hospital of Fujian University of Traditional Chinese Medicine (number: 2016KY-032-01). All participants provided informed, written consent before participation. Participants received an honorarium to acknowledge their research contribution.

### Participants

Patients were eligible to participate in the study if they met the following criteria: (1) aged at least 18 years, (2) diagnosis of first stroke, (3) normal speech function according to the Mandarin Language Screening Test with cutoff scores of >13 for those with primary school education or >14 for those with junior high school or higher education, (4) normal cognitive function according to the Montreal Cognitive Assessment with a cutoff score of >26, and (5) home discharge. Participants were excluded if they (1) did not own a mobile phone, (2) were unwilling to install and use the WeChat software on their mobile phone, (3) had emotional dysfunction according to the Beck Depression Inventory with a cutoff score of >13, or (4) had other medical illnesses limiting study participation. As we included only participants who served as their own informant rather than including participants on a nonselected basis, it is likely that individuals who were too cognitively impaired or were unable to understand the study materials were excluded.

### Recruitment and Screening

Participants were recruited from the inpatient rehabilitation hospital. Recruitment was initiated while the stroke patient was still in the hospital. The research assistant screened the medical records of all patients undergoing inpatient rehabilitation for a stroke. Once potential participants were identified, the research assistant approached the individual and provided information about the study. Participants provided informed consent once they agreed to participate. The research assistant reviewed the inclusion and exclusion criteria and completed the screening tests to confirm eligibility. Participants were then enrolled, and randomization occurred only after recruitment by a study coordinator.

### Data Collection Procedures

All research assistants received training in assessing the eligibility of potential participants, obtaining informed consent from participants, the study protocol, and obtaining outcome measures for both groups. They also received training in how to coach and assist participants using the WeChat app, including the videoconference and telephone call functions. After randomization, all participants received the baseline assessment at the week of discharge, followed by the completion of 2 mHealth app follow-up sessions (either videoconference or telephone), and half of the participants from each group received 2 home visits. The first follow-up session occurred 2 weeks after home discharge. Within 1 week of the first follow-up session, half of the study participants were selected to conduct the first home visit based on stratified sampling in each group. The stratified sampling criteria were grounded on participants’ functional abilities. As home visits are costly, this study was limited by randomly selecting half of the study participants for the home visit assessment. The second follow-up session occurred 3 months after home discharge. Within 1 week of the second follow-up session, we completed the second home visit in this subgroup of participants. The time interval between videoconference or telephone follow-up and home visit of 1 week was considered long enough to ensure that the previous responses were forgotten and short enough to ensure that the patient’s clinical condition would not substantially change. All assessments were conducted by our trained research assistants with training in physical therapy, occupational therapy, or rehabilitation medicine. To reduce assessor bias in the trial, research assistants who completed the videoconference or telephone follow-up sessions also conducted home visits with patients in the same group. We treated the face-to-face, home visit assessment as the gold standard in this study.

Participants in the videoconference follow-up group received training on the usage of the videoconference function of the WeChat app before discharge. During the follow-up sessions, research assistants asked participants to complete individual functional tasks and rate their actual performance through the videoconference, with the exception of bladder and bowel management tasks, which were assessed by the participant’s verbal descriptions. Participants also described difficulties pertaining to their individual task performance. During the home visit, research assistants completed the face-to-face observations by rating participants as they completed the same functional tasks. We used the same scoring criteria to evaluate the performance of our study participants in both videoconference and face-to-face, home visit assessments. Participants in the telephone follow-up group received training on the usage of the telephone function of the WeChat app before discharge. During the follow-up sessions, research assistants made telephone calls, asked participants how they performed in each functional item, and appraised their performance based on the participant’s verbal descriptions. During the home visit, research assistants completed the same protocol as in the videoconference follow-up group. We used the same scoring criteria to evaluate the performance of our study participants in both telephone and home visit assessments.

### Outcome Measures

As recommended in a systematic review of optimal outcome measures for stroke therapy trials [32], the primary outcome selected for this study was improvement in activities and participation rather than the reduction of impairments. Thus, we chose the functional status (ie, the performance of activities of daily living, ADLs) of stroke participants as our primary outcome variable. We used the Modified Barthel Index (MBI) to assess the performance of ADLs in the 2 groups [33,34]. The MBI can be administered using clinician-rated or patient-reported methods. It includes 10 items measuring grooming, bathing, feeding, toileting, stair climbing, dressing, bowel control, bladder control, mobility, and chair/bed transfer. Items have different response options, with anchored scores provided for different options. The total score ranges from 0 to 100. A higher score means that the participant has greater independence. Adequate validity and reliability were found for the Chinese version of the MBI used in this study [35].

We also defined 2 outcome variables to examine the feasibility of using an mHealth app to measure functional status of stroke participants in this study. The first variable was the completion of the MBI among our study participants in both groups at both follow-up periods. The second variable was the acceptability among our study participants (ie, levels of satisfaction, comfort, and confidence) of using the videoconference or telephone functions to complete the functional assessment at follow-up periods. We developed 3 questions: (1) “Are you satisfied with this follow-up assessment?” (2) “Are you comfortable with this follow-up assessment?” and (3) “Are you confident using this follow-up assessment?” All items were rated on a 4-point scale ranging from “very satisfied/comfortable/confident” to “unsatisfied/uncomfortable/unconfident.”

### Statistical Analysis

Data were analyzed using the Statistical Package for Social Sciences, version 20 (IBM, Chicago, IL, United States). Baseline characteristics between groups were compared using *t* tests or Mann-Whitney tests for continuous variables, and Pearson chi-square tests for categorical variables. We applied Wilcoxon signed-rank tests to compare MBI score differences between videoconference and telephone assessments, as well as between videoconference/telephone and home visit assessments for the validity evaluation. We set the *P* value to .05 for statistical significance. We used intraclass correlation coefficients (ICCs) to evaluate the agreement of all item scores between these assessments for the reliability evaluation. According to Landis and Koch [36], the ICC can theoretically vary between 0 and 1.0, where an ICC of 0 indicates no reliability, and an ICC of 1.0 indicates perfect reliability; ICCs above 0.80 indicate acceptable reliability. We used chi-square tests or Fisher exact tests to compare rates of completion, satisfaction, comfort, and confidence between the videoconference and telephone modes of administration.

## Results

### Baseline Characteristics

**Figure 1:**
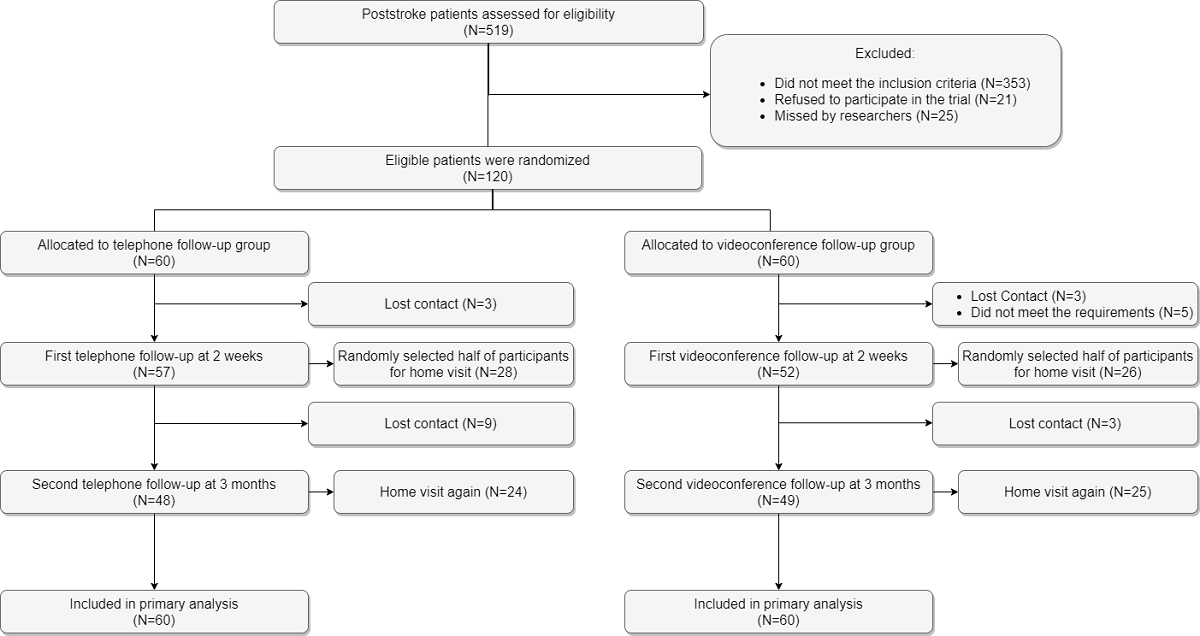
A flow diagram of patient enrollment.

Figure 1 illustrates the flow of participant enrollment. Among 519 potential stroke inpatients, 353 did not meet the inclusion criteria. A total of 21 patients refused to participate in the study because family members were uncertain about the use of mHealth for collecting data. Some patients reported that they could easily access medical services and did not require additional follow-up services. In total, 25 patients were discharged from the hospital before research assistants approached them. A total of 120 participants were successfully recruited and randomized to 1 of the 2 groups. Table 1 describes the demographic characteristics of study participants. Study participants were middle-aged (mean age 59.7 years, SD 12.1), 59.1% (71/120) of participants were women, and 93.3% (112/120) of participants were married. In total, 45.8% (55/120) of participants completed 9 or fewer years of formal education, and 28.3% (34/120) of participants were currently employed. A total of 45.0% (54/120) of participants had a history of cerebral infarction (ie, ischemic stroke). We found no significant differences between the 2 groups on gender, marital status, education, occupation, type of stroke, or duration of disease. We also found no significant differences between the 2 groups in any functional task measured by the MBI at the time of discharge. Eight participants in the videoconference follow-up group dropped out at 2 weeks: 3 participants did not answer the video calls and 5 participants refused to complete the assessment. Three participants in the telephone follow-up group dropped out at 2 weeks; they did not answer the telephone calls. At the 3-month follow-up point, we lost more participants: 3 participants in the videoconference follow-up group and 9 in the telephone follow-up group. Of the initial 60 participants in each group, 82% (49/60) of participants in the videoconference follow-up group and 80% (48/60) of participants in the telephone follow-up group completed the entire study protocol.

**Table 1:** Baseline characteristics of participants.

Variables		All participants (N=120)	Telephone follow-up (n=60)	Videoconference follow-up (n=60)	*Z* test or *Χ*^2^ (df) test		*P* value	
Age (years), mean (SD)		59.7 (12.1)	60.8 (11.6)	58.3 (12.4)	1.56		.25	
**Gender, n (%)**						**0.14 (1)**		**.85**
	Male	49 (40.8)	24 (41.7)	25（36.7）				
	Female	71 (59.1)	36（58.3）	35（63.3）				
**Marital status, n (%)**						**0.53 (1)**		**.72**
	Married	112 (93.3)	57（95.0）	55（91.7）				
	Other	8 (6.7)	3（5.0）	5（8.3）				
**Education (years), n (%)**						**1.38 (3)**		**.71**
	≤6	25 (20.8)	14（23.3）	11（18.3）				
	7-9	41 (34.2)	22（36.7）	19（31.7）				
	9-12	30 (25.0)	14（23.3）	16（26.7）				
	≥12	24 (20.0)	10（16.7）	14（23.3）				
**Occupation, n (%)**						**1.57 (3)**		**.67**
	Employed	34 (28.3)	19（31.7）	15（25.0）				
	Retired	50 (41.7)	26（43.3）	24（40）				
	Unemployed	10 (8.3)	4（6.7）	6（10）				
	Other	26 (21.7)	11（18.3）	15（25）				
**Type of stroke, n (%)**						**2.15 (1)**		**.14**
	Infarction	54 (45.0)	23（38.3）	31（51.7）				
	Hemorrhage	66 (55.0)	37（61.7）	29（48.3）				
Duration of stroke (days), mean (SD)		90.7 (13.8)	87.6 (14.6)	93.7 (12.9)	0.53		.24	
**Discharge functional status (Modified Barthel Index), mean (SD)**								
	Feeding	7.27 (2.09)	7.35 (1.92)	7.12 (2.08)	–0.59		.55	
	Grooming	3.06 (0.83)	3.02 (0.68)	3.15 (0.90)	–1.64		.10	
	Dressing	5.32 (2.10)	5.08 (2.09)	5.62 (2.27)	–1.35		.18	
	Bathing	2.02 (1.46)	2.23 (1.09)	1.85 (1.15)	–2.02		.06	
	Toilet use	5.21 (2.24)	5.13 (1.82)	5.32 (2.14)	–0.49		.62	
	Bowels	8.87 (1.64)	8.65 (1.63)	9.08 (1.33)	–1.30		.19	
	Bladder	9.06 (1.17)	9.00 (1.24)	9.18 (1.11)	–1.24		.22	
	Transfer	8.57 (2.89)	8.40 (2.68)	8.70 (3.03)	–0.68		.49	
	Mobility	7.73 (2.89)	8.15 (2.92)	7.42 (3.31)	–1.34		.18	
	Stairs	4.66 (2.17)	4.48 (1.87)	4.83 (2.27)	–0.77		.44	

### Comparison of Videoconference and Telephone Follow-Up Assessments

Table 2 shows the MBI scores for videoconference follow-up and telephone follow-up at 2-week and 3-month periods. We found no significant differences between the 2 groups in the majority of functional tasks measured by the MBI at 2 weeks and 3 months, except that significant differences were found in the bladder management task at 2 weeks, and the grooming and bathing tasks at 3 months. ICC values for all but the grooming task at 2 weeks and the grooming, toilet use, and mobility tasks at 3 months exceeded 0.8, indicating acceptable reliability between videoconference and telephone assessments at both follow-up periods.

**Table 2:** Comparison of Modified Barthel Index scores evaluated by videoconference and telephone follow-up assessments at 2-week follow-up and 3-month follow-up.

Variables		Telephone follow-up, mean (SD)	Videoconference follow-up, mean (SD)	*Z* test	*P* value	Intraclass correlation coefficient
**2-week follow-up**						
	Feeding	8.64 (1.59)	8.44 (1.72)	–1.64	.10	0.82
	Grooming	3.65 (0.69)	3.45 (0.76)	–1.79	.08	0.74
	Dressing	6.74 (2.00)	6.44 (2.03)	–1.90	.06	0.83
	Bathing	3.07 (1.14)	2.72 (1.22)	–1.38	.15	0.86
	Toilet use	7.13 (2.13)	6.28 (1.84)	–1.72	.07	0.81
	Bowels	8.63 (1.50)	9.03 (0.99)	–1.53	.12	0.88
	Bladder	9.04 (1.66)	9.45 (0.89)	–2.06	.04	0.83
	Transfer	9.50 (2.63)	9.85 (2.78)	–1.46	.13	0.82
	Mobility	8.75 (2.90)	8.25 (2.99)	–0.79	.42	0.85
	Stairs	6.67 (2.05)	6.28 (1.83)	–0.58	.56	0.81
**3-month follow-up**						
	Feeding	8.93 (1.59)	8.63 (1.38)	–1.77	.08	0.85
	Grooming	4.28 (0.80)	3.93 (0.58)	–2.85	.04	0.65
	Dressing	7.33 (2.28)	6.68 (1.81)	–1.86	.06	0.81
	Bathing	3.30 (0.99)	2.80 (1.09)	–1.93	.05	0.82
	Toilet use	7.25 (1.94)	6.78 (1.87)	–1.25	.21	0.76
	Bowels	9.23 (1.21)	9.43 (0.91)	–0.70	.48	0.81
	Bladder	9.03 (1.59)	9.50 (0.87)	–1.29	.20	0.83
	Transfer	10.38 (2.74)	10.47 (2.70)	–0.32	.75	0.82
	Mobility	10.32 (3.13)	9.45 (2.99)	–1.49	.14	0.66
	Stairs	7.05 (2.05)	6.48 (2.33)	–1.33	.18	0.80

### Comparison of Two Mobile Health Follow-Up Assessments With Home Visit Assessments

#### Selecting Candidates for Home Visit Assessments in Two Groups

We adopted stratified sampling in each group to select subgroups of participants for home visit assessments. We used the discharge MBI scores of 52 participants in the videoconference follow-up group and 57 participants in the telephone follow-up group to classify their functional independence levels into 4 videoconference follow-up subgroups and 4 telephone follow-up subgroups, respectively. The number of participants in the videoconference follow-up and telephone follow-up subgroups is shown in Table 3. We selected half of the participants in each of the 8 subgroups for home visit assessments.

**Table 3:** Distribution of discharge Modified Barthel Index scores in the videoconference follow-up and telephone follow-up subgroups.

MBI^a^ scores	Video follow-up, n (%)	Telephone follow-up, n (%)
Complete dependence (MBI<40)	3 (5.8)	4 (7)
Dependence (MBI 40-59)	17 (32.7)	16 (28)
Mild dependence (MBI 60-99)	30 (57.7)	36 (63.1)
Independence (MBI 100)	2 (3.8)	1 (1.9)

^a^MBI: Modified Barthel Index.

#### Comparison of Modified Barthel Index Scores Between Videoconference Follow-Up and Home Visit

Table 4 shows the MBI scores for videoconference follow-up and face-to-face, home visit assessments at 2-week follow-up. MBI scores collected by videoconference were similar to those collected by the face-to-face, home visit assessment, except that a significant difference was found in the feeding task. We also found that ICC values for all 10 tasks were above 0.8, indicating acceptable reliability between the 2 assessments at 2-week follow-up. Table 4 also shows the MBI scores for videoconference follow-up and home visit assessments at 3-month follow-up. Similar comparison results were found between the 2 assessments at 3 months, except that a significant difference was found in the transfer task. ICC values for all but the transfer and stair-climbing tasks exceeded 0.8, indicating acceptable reliability between videoconference and home visit assessments at 3 months.

**Table 4:** A comparison of MBI scores evaluated in videoconference and home visit assessments at 2-week follow-up (n=26) and 3-month follow-up (n=25).

Variables		Videoconference, mean (SD)		Home visit, mean (SD)		*Z* test		*P* value		Intraclass correlation coefficient	
**2-week follow-up**											
	Feeding		8.04 (1.75)		7.23 (1.95)		−2.60		<.001		0.87
	Grooming		3.30 (0.88)		3.50 (0.76)		−1.89		.06		0.90
	Dressing		6.35 (2.21)		6.58 (2.21)		−1.00		.32		0.92
	Bathing		2.46 (1.27)		2.54 (1.24)		0.49		.62		0.87
	Toilet use		6.23 (2.02)		5.77 (2.08)		−1.63		.10		0.87
	Bowels		9.30 (0.97)		9.23 (0.99)		−0.58		.56		0.86
	Bladder		9.46 (0.90)		9.36 (0.96)		−0.09		.87		0.89
	Transfer		9.27 (2.84)		9.15 (3.51)		−0.72		.47		0.84
	Mobility		8.96 (3.18)		9.30 (2.99)		1.0		.32		0.87
	Stairs		6.42 (1.96)		5.81 (1.83)		−1.83		.07		0.82
**3-month follow-up**											
	Feeding		8.54 (1.42)		8.12 (1.80)		–1.73		.08		0.83
	Grooming		4.04 (0.53)		4.12 (0.65)		–1.00		.32		0.88
	Dressing		6.77 (1.75)		7.04 (1.56)		–1.51		.13		0.92
	Bathing		2.77 (1.10)		3.00 (1.13)		–1.35		.18		0.82
	Toilet use		6.77 (1.95)		7.00 (1.90)		0.81		.42		0.80
	Bowels		9.31 (0.97)		9.38 (0.94)		–0.58		.56		0.89
	Bladder		9.46 (0.90)		9.54 (0.86)		–1.00		.32		0.95
	Transfer		10.77 (2.80)		9.50 (3.31)		–2.41		.02		0.75
	Mobility		9.81 (2.23)		9.88 (2.76)		0.14		.89		0.85
	Stairs		6.88 (2.10)		6.35 (1.65)		–1.48		.14		0.76

### Comparison of Modified Barthel Index Scores Between Telephone Follow-Up and Home Visit

Table 5 shows the MBI scores for telephone follow-up and face-to-face, home visit assessments at 2-week follow-up. A comparison of these assessments found that almost all MBI scores collected by telephone administration were statistically higher than those collected by the home visit assessment, with the exception of bowel and bladder management tasks, indicating that the telephone administration method may have overestimated the functional status of study participants for most tasks. ICC values indicate that inadequate reliability was found between the 2 assessment methods at 2 weeks; 8 out of 10 tasks had ICC values less than 0.8.

Table 5 also shows the MBI scores for telephone follow-up and home visit assessments at 3-month follow-up. In general, MBI scores for telephone follow-up were slightly higher than those for home visit assessment, but the only significant differences were found for 4 tasks: feeding, grooming, bathing, and stair climbing. Eight tasks had ICC values less than 0.8, indicating that inadequate reliability was found between the 2 assessment methods at 3 months. Compared with the results of the 2-week follow-up, ICC values showed a general downward trend at the 3-month follow-up.

**Table 5:** Comparison of Modified Barthel Index scores evaluated in telephone and home visit assessments at 2-week follow-up (n=28) and 3-month follow-up (n=24).

Variables		Telephone, mean (SD)	Home visit, mean (SD)	*Z* test	*P* value	Intraclass correlation coefficient
**2-week follow-up**						
	Feeding	8.39 (1.85)	6.79 (1.99)	−3.22	<.001	0.66
	Grooming	3.68 (0.72)	3.36 (0.78)	−1.97	.05	0.58
	Dressing	6.82 (2.00)	5.71 (2.02)	−2.52	.01	0.64
	Bathing	3.07 (1.15)	2.17 (1.19)	-3.15	<.001	0.65
	Toilet use	7.53 (2.11)	5.25 (2.19)	−3.63	<.001	0.62
	Bowels	8.54 (1.57)	8.32 (1.83)	−1.03	.31	0.82
	Bladder	8.86 (1.80)	8.82 (1.80)	−0.30	.76	0.80
	Transfer	10.60 (2.47)	8.64 (3.23)	−3.07	<.001	0.68
	Mobility	10.93 (2.97)	8.36 (3.23)	−3.21	<.001	0.63
	Stairs	7.29 (1.88)	5.61 (1.79)	−3.31	<.001	0.61
**3-month follow-up**						
	Feeding	9.00 (1.53)	7.37 (2.10)	−3.04	<.001	0.62
	Grooming	4.46 (0.72)	4.12 (0.61)	−2.20	.05	0.52
	Dressing	7.58 (2.22)	6.83 (2.18)	−1.50	.13	0.63
	Bathing	3.50 (0.98)	2.92 (1.02)	−2.08	.04	0.49
	Toilet use	7.17 (2.01)	6.58 (1.89)	−1.15	.25	0.58
	Bowels	9.20 (1.28)	9.04 (1.30)	−0.82	.41	0.82
	Bladder	9.08 (1.53)	9.13 (1.53)	−0.33	.74	0.81
	Transfer	10.54 (2.57)	9.79 (3.44)	−1.32	.19	0.66
	Mobility	10.37 (2.93)	9.42 (3.09)	−1.41	.16	0.62
	Stairs	7.29 (1.83)	6.54 (1.79)	−1.73	.08	0.49

### Feasibility of Using the Videoconference and Telephone Function for Collecting Follow-Up Data

As shown in Figure 1 and Table 6, 8 out of 60 (13%) participants in the videoconference follow-up group dropped out, and 3 out of 60 (5%) participants in the telephone follow-up group dropped out at 2-week follow-up. There was no significant difference in completion rates between the 2 groups (*Χ*^2^_1_=1.6; *P*=.21) at 2 weeks. At 3-month follow-up, 3 out of 52 (6%) participants in the videoconference follow-up group dropped out, and 9 out of 57 (16%) participants in the telephone follow-up group dropped out. There was no significant difference in the completion rates between the 2 groups (*Χ*^2^_1_=1.86; *P*=.17) at 3 months.

**Table 6:** Completion rates at 2-week and 3-month follow-up assessments.

Completion rates	Videoconference follow-up	Telephone follow-up	*Χ*^2^ (df)	*P* value
Completion (2-week follow-up)	52	57	1.60 (1)	.21
Dropout (2-week follow-up)	8	3	N/A^a^	N/A
Completion (3-month follow-up)	49	48	1.86 (1)	.17
Dropout (3-month follow-up)	3	9	N/A	N/A

^a^N/A: not applicable.

Table 7 shows participant ratings of satisfaction, comfort, and confidence with using either the videoconference or telephone call function for follow-up assessments. At 2-week follow-up, the majority of participants were either very satisfied (22/52, 42%) or satisfied (29/52, 56%) with the videoconference function, and either very satisfied (19/57, 33%) or satisfied (34/57, 60%) with the telephone call function. There was no significant difference in the satisfaction levels between the 2 groups (*Χ*^2^_3_=2.5; *P*=.28) at 2 weeks. At 3-month follow-up, participants in the videoconference follow-up group reported higher satisfaction than those in the telephone follow-up group (*Χ*^2^_3_=13.9; *P*=.03). Additionally, most participants were either very comfortable (24/52, 46% at 2 weeks; 26/48, 54% at 3 months) or comfortable (28/52, 54% at 2 weeks; 20/48, 42% at 3 months) with the videoconference function, and most participants were either very comfortable (33/57, 58% at 2 weeks; 21/49, 43% at 3 months) or comfortable (24/57, 42% at 2 weeks; 26/49, 53% at 3 months) with the telephone call function. There were no differences in comfort levels between the 2 groups at 2 weeks (*Χ*^2^_3_=1.5; *P*=.22) or 3 months (*Χ*^2^_3_=1.3; *P*=.52). Regarding participant confidence using the videoconference or telephone function for collecting functional data, participants in the videoconference follow-up group rated higher confidence than those in the telephone follow-up group at 2 weeks (*Χ*^2^_3_=6.6; *P*=.04) and 3 months (*Χ*^2^_3_=7.9; *P*=.04).

**Table 7:** Ratings of satisfaction, comfort, and confidence at 2-week and 3-month follow-up assessments.

Ratings		Videoconference follow-up, n (%)		Telephone follow-up, n (%)		*Χ*^2^ (df)		*P* value	
**Satisfaction (2-week follow-up)**						**2.54 (3)**		**.28**	
	Very satisfied		22 (42)		19 (33)				
	Satisfied		29 (56)		34 (60)				
	Not very satisfied		1 (2)		4 (7)				
	Unsatisfied		0 (0)		0 (0)				
**Satisfaction (3-month follow-up)**						**13.9 (3)**		**.03**	
	Very satisfied		30 (61)		12 (25)				
	Satisfied		17 (35)		30 (63)				
	Not very satisfied		2 (4)		4 (8)				
	Unsatisfied		0 (0)		2 (4)				
**Comfort (2-week follow-up)**						**1.50 (3)**		**.22**	
	Very comfortable		24 (46)		33 (58)				
	Comfortable		28 (54)		24 (42)				
	Not very comfortable		0 (0)		0 (0)				
	Uncomfortable		0 (0)		0 (0)				
**Comfort (3-month follow-up)**						**1.30 (3)**		**.52**	
	Very comfortable		26 (54)		21 (43)				
	Comfortable		20 (42)		26 (53)				
	Not very comfortable		2 (4)		2 (4)				
	Uncomfortable		0 (0)		0 (0)				
**Confidence (2-week follow-up)**						**6.68 (3)**		**.04**	
	Very confident		33 (63)		24 (42)				
	Confident		19 (37)		30 (53)				
	Not very confident		0 (0)		3 (5)				
	Unconfident		0 (0)		0 (0)				
**Confidence (3-month follow-up)**						**7.97 (3)**		**.04**	
	Very confident		31 (63)		20 (42)				
	Confident		18 (37)		23 (48)				
	Not very confident		0 (0)		2 (4)				
	Unconfident		0 (0)		3 (6)				

## Discussion

This is one of the first studies to compare the validity and reliability of the videoconference and telephone functions of an mHealth app for collecting functional status data in stroke patients after rehabilitation hospitalization and to examine the feasibility and acceptability of using these modes of administration for data collection. We examined these questions prospectively in a cohort of patients who were discharged from inpatient stroke rehabilitation by comparing videoconference and telephone follow-up assessments, as well as comparing these 2 modes of administration to the home visit assessment as the gold standard.

### Validity and Reliability of the Mobile Health App for Collecting Functional Status Data

We found that most MBI scores obtained by videoconference administration were slightly lower than those obtained by telephone administration, although these score differences did not achieve statistical significance at either 2-week or 3-month periods. Our findings further revealed that videoconference, but not telephone, administration was as valid and reliable as face-to-face, home visit assessment at both 2-week and 3-month follow-up periods. Home visit assessment is conventionally regarded as one of the best methods for collecting posthospitalization outcome measurement data [37]. Home visit (or home rehabilitation) is recognized as providing greater convenience to patients and families while encouraging therapy to continue to occur within the patient’s home; however, it is less cost-effective [38]. One of the reasons for this is that home therapists can often only visit one patient at a time. A previous rehabilitation study indicated that the mean amount of time allotted to perform assessments in home visits is 1 hour and 57 minutes (SD 19 minutes) [39]. Additional time is needed to travel to the patient’s home, which can be even more time consuming for patients who live at a greater distance. Furthermore, inadequate manpower and financial concerns restrict the implementation of home visit assessments for all patients after hospitalization. Instead, other studies [40] have recommended home-based telemedicine as a viable option in the delivery of poststroke recovery programs, because telemedicine has shown promising results in improving the overall health of stroke patients and in supporting caregivers while being delivered by therapists from a distance. The use of technologies appears to be a novel potential approach for the therapist’s assessment of patient performance in home settings. Our findings concur with this notion that an app-based videoconference can be used to assess the functional performance of stroke patients in home settings. The videoconference function may augment other technology-enabled solutions to provide a means of conducting future clinical trials aimed to evaluate the outcomes of any rehabilitation program implemented in the patient’s home [41,42].

Our findings revealed that almost all MBI scores obtained by telephone administration were higher than those obtained via home visit. This overestimation of functional scores is particularly obvious at 2-week follow-up; telephone assessment of functional status using the MBI was less reliable compared with the home visit at 2-week follow-up, but reliability did improve at 3-month follow-up. Previous studies have attained strong agreement between telephone and face-to-face assessments [43,44]. Psychometric differences may be partially attributed to the use of diverse scales in different studies. Our study used the MBI, whereas 2 other studies used either the Functional Independence Measure or Modified Rankin Scales to measure poststroke disability. Interestingly, Pietra et al [45] conducted a similar study to compare the validity and reliability of the Barthel Index (BI) administered by telephone compared with face-to-face assessment in patients after stroke. They indicated that telephone assessment with the BI is reliable in comparison with face-to-face assessment. Several possible reasons could explain these differences. First, the measurement tool used in our study was the MBI, which is more rigorous and provides more detailed ratings compared with the BI. Second, the stroke sample in our study consisted of individuals who were discharged from the rehabilitation hospital, whereas the stroke sample in their study consisted of inpatients in the hospital. The use of telephone interview is greatly contingent upon whether patients are cognizant of their self-function. In our study, discharged patients who were living in their home and community settings were more likely to experience their actual function, as they have more opportunities to interact with real-world contextual barriers. This view is further supported by our study findings, wherein we found greater agreement between 3-month telephone and home visit assessments than 2-week telephone and home visit assessments; individuals at the 3-month follow-up point have had more exposure to their real-world environmental barriers and are better able to estimate their functional status.

### Feasibility of the Mobile Health App for Collecting Functional Status Data

It is noteworthy that our findings indicate that completion rates of both videoconference and telephone assessments were greater than 80% at all follow-up periods. These completion rates are within the acceptable range in clinical studies [46]. Moreover, compared with the telephone assessment, patients reported higher satisfaction with and confidence using the videoconference assessment to measure their functional status. A similar study with the WeChat app for health education also revealed high satisfaction perceived by their participants [47]. All of these results confirm that videoconference assessment of the MBI administered via the WeChat app can serve as an alternative tool to the face-to-face, home visit assessment. Videoconference follow-up provides a surveillance platform for clinicians to objectively assess the task performance of patients in their homes. Patients may also perceive a strong sense of participation, which can improve their psychological condition [48]. Prior research [31] and our results have demonstrated the beneficial effects of the WeChat app as a time-effective, cost-effective, and acceptable communication tool for follow-up data collection. However, implementing routine follow-up measurement via technology involves a number of considerations, including the selection of appropriate patients, settings, timing of assessment, and the optimal mode of administration [49]. Offering different modes of administration, such as video consultation, voice communication, text messaging, or image sharing, may help minimize biased sampling and increase patient participation. Future research may consider adopting a mixed method approach that could help to identify facilitators and barriers to the adoption of mHealth apps for collecting posthospitalization data. In addition, the choice of modalities for monitoring patients depends on the size and structure of the organization. An earlier study [50] found that larger organizations report fewer barriers to using technology-based therapeutic tools, likely due to greater resources. Thus, from a researcher or service provider standpoint, this system-level factor is equally important in determining the best mode of administration for follow-up data collection after discharge from inpatient rehabilitation.

### Study Strengths and Limitations

Our study has a number of strengths. Prior research testing the feasibility and psychometrics of mHealth assessments has primarily adopted the observational design, but ours is one of the first few studies to employ the randomized controlled design to compare these characteristics for 2 different mHealth methods to collect outcome data after discharge from inpatient stroke rehabilitation. Additionally, this study conducted 2 follow-up sessions for both assessment methods to better understand any issues related to long-term compliance. Yet, our study has several limitations. First, patients were recruited from one rehabilitation hospital in a coastal province in eastern China; therefore, results may not generalize to persons living in other regions. Second, this study only included participants who owned a mobile phone and served as their own informant to complete the assessment; patients without a mobile phone and those with severe cognitive or communication impairments may have been excluded from this study. Third, the MBI was the only outcome assessment used in the study. Future research should explore mHealth assessments for measuring other health outcomes in poststroke individuals after discharge from the hospital. Another limitation includes measuring acceptability through 3 self-constructed items (ie, satisfaction, comfort, and confidence), which are limited in their measurement of this construct and do not provide actionable data with which to inform future research. Even though these self-constructed items allow for efficient quantification of acceptability, future research should add a qualitative or mixed method approach to provide additional interpretation and meaning to the quantitative results [51]. Furthermore, this study did not record the amount of time required for performing videoconference and telephone follow-up assessments. Future research should measure the duration of these 2 follow-up assessments and compare them with the time needed for the home visit assessment to provide additional validation evidence. Future research is also needed to conduct a randomized controlled trial comparing these 2 modes of mobile administration to traditional telephone interview method (eg, landline phone service) for stroke patients as a control condition.

### Conclusions

This study found satisfactory feasibility and validity of an app-based videoconference method for collecting functional data after inpatient stroke rehabilitation. High completion and acceptability, as well as adequate validity and reliability of the videoconference follow-up method, may support its clinical application in poststroke home rehabilitation programs and long-term health monitoring after hospitalization.

## Appendix

Multimedia Appendix 1 CONSORT-eHEALTH checklist (V 1.6.1).

## Abbreviations

ADLs: activities of daily living
BI: Barthel Index
ICC: intraclass correlation coefficient
MBI: Modified Barthel Index
mHealth: mobile health
