# Supplementary material for: A Mobile Health App for the Collection of Functional Outcomes After Inpatient Stroke Rehabilitation: Pilot Randomized Controlled Trial
Source: JMIR Mhealth Uhealth. 2020 May 13;8(5):e17219. doi: 10.2196/17219 (PMC7254286; doi:10.2196/17219)
Supplement: Multimedia Appendix 1 [file mhealth_v8i5e17219_app1.pdf]

# CONSORT-EHEALTH (V 1.6.1) - Submission/Publication Form

The CONSORT-EHEALTH checklist is intended for authors of randomized trials evaluating web-based and Internet-based applications/interventions, including mobile interventions, electronic games (incl multiplayer games), social media, certain telehealth applications, and other interactive and/or networked electronic applications. Some of the items (e.g. all subitems under item 5 - description of the intervention) may also be applicable for other study designs.

The goal of the CONSORT EHEALTH checklist and guideline is to be
a) a guide for reporting for authors of RCTs,
b) to form a basis for appraisal of an ehealth trial (in terms of validity)

CONSORT-EHEALTH items/subitems are MANDATORY reporting items for studies published in the Journal of Medical Internet Research and other journals / scientific societies endorsing the checklist.

Items numbered 1., 2., 3., 4a., 4b etc are original CONSORT or CONSORT-NPT (non-pharmacologic treatment) items.
Items with Roman numerals (i., ii, iii, iv etc.) are CONSORT-EHEALTH extensions/clarifications.

As the CONSORT-EHEALTH checklist is still considered in a formative stage, we would ask that you also RATE ON A SCALE OF 1-5 how important/useful you feel each item is FOR THE PURPOSE OF THE CHECKLIST and reporting guideline (optional).

Mandatory reporting items are marked with a red *.
In the textboxes, either copy & paste the relevant sections from your manuscript into this form - please include any quotes from your manuscript in QUOTATION MARKS,
or answer directly by providing additional information not in the manuscript, or elaborating on why the item was not relevant for this study.

YOUR ANSWERS WILL BE PUBLISHED AS A SUPPLEMENTARY FILE TO YOUR PUBLICATION IN JMIR AND ARE CONSIDERED PART OF YOUR PUBLICATION (IF ACCEPTED).
Please fill in these questions diligently. Information will not be copyedited, so please use proper spelling and grammar, use correct capitalization, and avoid abbreviations.

DO NOT FORGET TO SAVE AS PDF _AND_ CLICK THE SUBMIT BUTTON SO YOUR ANSWERS ARE IN OUR DATABASE !!!

Citation Suggestion (if you append the pdf as Appendix we suggest to cite this paper in the caption):
Eysenbach G, CONSORT-EHEALTH Group
CONSORT-EHEALTH: Improving and Standardizing Evaluation Reports of Web-based and Mobile Health Interventions
J Med Internet Res 2011;13(4):e126
URL: http://www.jmir.org/2011/4/e126/

doi: 10.2196/jmir.1923
PMID: 22209829

*必填

Your name *

First Last

Alex Wong

Primary Affiliation (short), City, Country *

University of Toronto, Toronto, Canada

Washington University School of Medicine, St.

Your e-mail address *

abc@gmail.com

wongal@wustl.edu

Title of your manuscript *

Provide the (draft) title of your manuscript.

Mobile Health Application for Collection of Functional Outcomes after Inpatient Stroke Rehabilitation: Pilot Randomized Controlled Trial

Name of your App/Software/Intervention *

If there is a short and a long/alternate name, write the short name first and add the long name in brackets.

WeChat (Chinese version: Weixin)

Evaluated Version (if any)

e.g. "V1", "Release 2017-03-01", "Version 2.0.27913"

您的回答

Language(s) *

What language is the intervention/app in? If multiple languages are available, separate by comma (e.g. "English, French")

WeChat (Chinese version: Weixin)

URL of your Intervention Website or App

e.g. a direct link to the mobile app on app in appstore (itunes, Google Play), or URL of the website. If the intervention is a DVD or hardware, you can also link to an Amazon page.

https://www.wechat.com/en/

URL of an image/screenshot (optional)

您的回答

Accessibility *

Can an enduser access the intervention presently?

- (selected) access is free and open
- access only for special usergroups, not open
- access is open to everyone, but requires payment/subscription/in-app purchases
- app/intervention no longer accessible
- 其他:

### Primary Medical Indication/Disease/Condition *

e.g. "Stress", "Diabetes", or define the target group in brackets after the condition, e.g. "Autism (Parents of children with)", "Alzheimers (Informal Caregivers of)"

Stroke

### Primary Outcomes measured in trial *

comma-separated list of primary outcomes reported in the trial

Modified Barthel Index (A measure of function

### Secondary/other outcomes

Are there any other outcomes the intervention is expected to affect?

您的回答

### Recommended "Dose" *

What do the instructions for users say on how often the app should be used?

- ○ Approximately Daily
- ○ Approximately Weekly
- ○ Approximately Monthly
- ○ Approximately Yearly
- ● "as needed"
- ○ 其他:

Approx. Percentage of Users (starters) still using the app as recommended after 3 months *

- ( ) unknown / not evaluated
- ( ) 0-10%
- ( ) 11-20%
- ( ) 21-30%
- ( ) 31-40%
- ( ) 41-50%
- ( ) 51-60%
- ( ) 61-70%
- (•) 71%-80%
- ( ) 81-90%
- ( ) 91-100%
- ( ) 其他:

Overall, was the app/intervention effective? *

- (•) yes: all primary outcomes were significantly better in intervention group vs control
- ( ) partly: SOME primary outcomes were significantly better in intervention group vs control
- ( ) no statistically significant difference between control and intervention
- ( ) potentially harmful: control was significantly better than intervention in one or more outcomes
- ( ) inconclusive: more research is needed
- ( ) 其他:

## Article Preparation Status/Stage *

At which stage in your article preparation are you currently (at the time you fill in this form)

- ○ not submitted yet - in early draft status
- ○ not submitted yet - in late draft status, just before submission
- ○ submitted to a journal but not reviewed yet
- ● submitted to a journal and after receiving initial reviewer comments
- ○ submitted to a journal and accepted, but not published yet
- ○ published
- ○ 其他:

## Journal *

If you already know where you will submit this paper (or if it is already submitted), please provide the journal name (if it is not JMIR, provide the journal name under "other")

- ○ not submitted yet / unclear where I will submit this
- ● Journal of Medical Internet Research (JMIR)
- ○ JMIR mHealth and UHealth
- ○ JMIR Serious Games
- ○ JMIR Mental Health
- ○ JMIR Public Health
- ○ JMIR Formative Research
- ○ Other JMIR sister journal
- ○ 其他:

Is this a full powered effectiveness trial or a pilot/feasibility trial? *

- (●) Pilot/feasibility
- ( ) Fully powered

Manuscript tracking number *

If this is a JMIR submission, please provide the manuscript tracking number under "other" (The ms tracking number can be found in the submission acknowledgement email, or when you login as author in JMIR. If the paper is already published in JMIR, then the ms tracking number is the four-digit number at the end of the DOI, to be found at the bottom of each published article in JMIR)

- ( ) no ms number (yet) / not (yet) submitted to / published in JMIR
- (●) 其他: ms #17219

## TITLE AND ABSTRACT

## 1a) TITLE: Identification as a randomized trial in the title

1a) Does your paper address CONSORT item 1a? *

I.e does the title contain the phrase "Randomized Controlled Trial"? (if not, explain the reason under "other")

- (●) yes
- ( ) 其他:

## 1a-i) Identify the mode of delivery in the title

Identify the mode of delivery. Preferably use “web-based” and/or “mobile” and/or “electronic game” in the title. Avoid ambiguous terms like “online”, “virtual”, “interactive”. Use “Internet-based” only if Intervention includes non-web-based Internet components (e.g. email), use “computer-based” or “electronic” only if offline products are used. Use “virtual” only in the context of “virtual reality” (3-D worlds). Use “online” only in the context of “online support groups”. Complement or substitute product names with broader terms for the class of products (such as “mobile” or “smart phone” instead of “iphone”), especially if the application runs on different platforms.

| | 1 | 2 | 3 | 4 | 5 | |
|---|---|---|---|---|---|---|
| subitem not at all important | ○ | ○ | ○ | ◉ | ○ | essential |

## Does your paper address subitem 1a-i? *

Copy and paste relevant sections from manuscript title (include quotes in quotation marks "like this" to indicate direct quotes from your manuscript), or elaborate on this item by providing additional information not in the ms, or briefly explain why the item is not applicable/relevant for your study

We used the term "Mobile" to describe the Wechat - the most common social software app in China - for this manuscript, like this "WeChat (Chinese version: Weixin), developed in 2011 by Tencent, has become the most common social software app in China [25]. " as well as like this "After participants provided informed consent and were screened for eligibility, participants were randomized into 1 of the 2 WeChat app administration groups: videoconference follow-up and telephone follow-up, with a

## 1a-ii) Non-web-based components or important co-interventions in title

Mention non-web-based components or important co-interventions in title, if any (e.g., “with telephone support”).

| | 1 | 2 | 3 | 4 | 5 | |
|---|---|---|---|---|---|---|
| subitem not at all important | ○ | ○ | ○ | ◉ | ○ | essential |

### Does your paper address subitem 1a-ii?

Copy and paste relevant sections from manuscript title (include quotes in quotation marks "like this" to indicate direct quotes from your manuscript), or elaborate on this item by providing additional information not in the ms, or briefly explain why the item is not applicable/relevant for your study

We used "telephone follow-up" method as our control arm, like this "A randomized controlled trial was conducted in a rehabilitation hospital in Southeast China. Participants were randomly assigned to either a videoconference follow-up or a telephone followup group. ", and like this "Our second aim was to examine the feasibility of the functional assessment administered via videoconference compared to telephone call in stroke patients after rehabilitation hospitalization. "

### 1a-iii) Primary condition or target group in the title

Mention primary condition or target group in the title, if any (e.g., “for children with Type I Diabetes”) Example: A Web-based and Mobile Intervention with Telephone Support for Children with Type I Diabetes: Randomized Controlled Trial

| | 1 | 2 | 3 | 4 | 5 | |
|---|---|---|---|---|---|---|
| subitem not at all important | ○ | ○ | ○ | ○ | ◉ | essential |

### Does your paper address subitem 1a-iii? *

Copy and paste relevant sections from manuscript title (include quotes in quotation marks "like this" to indicate direct quotes from your manuscript), or elaborate on this item by providing additional information not in the ms, or briefly explain why the item is not applicable/relevant for your study

We described "Stroke" in our title, like this "Mobile Health Application for Collection of Functional Outcomes after Inpatient Stroke Rehabilitation: Randomized Controlled

### 1b) ABSTRACT: Structured summary of trial design, methods, results, and conclusions

NPT extension: Description of experimental treatment, comparator, care providers, centers, and blinding status.

### 1b-i) Key features/functionalities/components of the intervention and comparator in the METHODS section of the ABSTRACT

Mention key features/functionalities/components of the intervention and comparator in the abstract. If possible, also mention theories and principles used for designing the site. Keep in mind the needs of systematic reviewers and indexers by including important synonyms. (Note: Only report in the abstract what the main paper is reporting. If this information is missing from the main body of text, consider adding it)

| | 1 | 2 | 3 | 4 | 5 | |
|---|---|---|---|---|---|---|
| subitem not at all important | ○ | ○ | ○ | ◉ | ○ | essential |

### Does your paper address subitem 1b-i? *

Copy and paste relevant sections from the manuscript abstract (include quotes in quotation marks "like this" to indicate direct quotes from your manuscript), or elaborate on this item by providing additional information not in the ms, or briefly explain why the item is not applicable/relevant for your study

We described about the components of the intervention and comparator in the abstract, like this "A randomized controlled trial was conducted in a rehabilitation hospital in Southeast China. Participants were randomly assigned to either a videoconference follow-up (n=60) or a telephone follow-up (n=60) group. We measured the functional status of participants in each group at 2-week and 3-month follow-up periods. Half of the participants in each group were followed by face-to-face, home visit assessments as the gold standard. Validity was assessed by comparing any score differences between videoconference follow-up and home visit assessments, as well as telephone follow-up and home visit assessments. Reliability was assessed by computing agreements between videoconference follow-up and home visit assessments, as well as telephone follow-up and home visit assessments. Feasibility was evaluated by levels of completion, satisfaction, comfort, and confidence in the 2 groups. " Our study did not include any testing of theories. Instead, we conducted a trial that compared different modes of administration for gathering functional outcomes of post-stroke patients after hospitalization.

### 1b-ii) Level of human involvement in the METHODS section of the ABSTRACT

Clarify the level of human involvement in the abstract, e.g., use phrases like “fully automated” vs. “therapist/nurse/care provider/physician-assisted” (mention number and expertise of providers involved, if any). (Note: Only report in the abstract what the main paper is reporting. If this information is missing from the main body of text, consider adding it)

| | 1 | 2 | 3 | 4 | 5 | |
|---|---|---|---|---|---|---|
| subitem not at all important | ○ | ○ | ○ | ◉ | ○ | essential |

### Does your paper address subitem 1b-ii?

Copy and paste relevant sections from the manuscript abstract (include quotes in quotation marks "like this" to indicate direct quotes from your manuscript), or elaborate on this item by providing additional information not in the ms, or briefly explain why the item is not applicable/relevant for your study

We described our trial including the face-to-face, home visit assessment to indicate that the therapist was involved in the trial, like this "We measured the functional status of participants in each group at 2-week and 3-month follow-up periods. Half of the participants in each group were followed by face-to-face, home visit assessments as the gold standard. "

### 1b-iii) Open vs. closed, web-based (self-assessment) vs. face-to-face assessments in the METHODS section of the ABSTRACT

Mention how participants were recruited (online vs. offline), e.g., from an open access website or from a clinic or a closed online user group (closed usergroup trial), and clarify if this was a purely web-based trial, or there were face-to-face components (as part of the intervention or for assessment). Clearly say if outcomes were self-assessed through questionnaires (as common in web-based trials). Note: In traditional offline trials, an open trial (open-label trial) is a type of clinical trial in which both the researchers and participants know which treatment is being administered. To avoid confusion, use “blinded” or “unblinded” to indicated the level of blinding instead of “open”, as “open” in web-based trials usually refers to “open access” (i.e. participants can self-enrol). (Note: Only report in the abstract what the main paper is reporting. If this information is missing from the main body of text, consider adding it)

| | 1 | 2 | 3 | 4 | 5 | |
|---|---|---|---|---|---|---|
| subitem not at all important | ○ | ○ | ○ | ◉ | ○ | essential |

### Does your paper address subitem 1b-iii?

Copy and paste relevant sections from the manuscript abstract (include quotes in quotation marks "like this" to indicate direct quotes from your manuscript), or elaborate on this item by providing additional information not in the ms, or briefly explain why the item is not applicable/relevant for your study

We describe our participants recruited from a rehabilitation hospital, like this "A randomized controlled trial was conducted in a rehabilitation hospital in Southeast China." We also described our trial including the face-to-face components as part of the assessment, like this "We measured the functional status of participants in each group at 2week and 3-month follow-up periods. Half of the participants in each group were followed by face-to-face, home visit assessments as the gold standard. "

## 1b-iv) RESULTS section in abstract must contain use data

Report number of participants enrolled/assessed in each group, the use/uptake of the intervention (e.g., attrition/adherence metrics, use over time, number of logins etc.), in addition to primary/secondary outcomes. (Note: Only report in the abstract what the main paper is reporting. If this information is missing from the main body of text, consider adding it)

| | 1 | 2 | 3 | 4 | 5 | |
|---|---|---|---|---|---|---|
| subitem not at all important | ○ | ○ | ○ | ● | ○ | essential |

## Does your paper address subitem 1b-iv?

Copy and paste relevant sections from the manuscript abstract (include quotes in quotation marks "like this" to indicate direct quotes from your manuscript), or elaborate on this item by providing additional information not in the ms, or briefly explain why the item is not applicable/relevant for your study

We reported the number of participants assessed in each group, like this "Participants were randomly assigned to either a videoconference follow-up (n=60) or a telephone follow-up ( n=60) group. We measured the functional status of participants in each group at 2week and 3-month follow-up periods. Half of the participants in each group were followed by face-to-face, home visit assessments as the gold standard. "

## 1b-v) CONCLUSIONS/DISCUSSION in abstract for negative trials

Conclusions/Discussions in abstract for negative trials: Discuss the primary outcome - if the trial is negative (primary outcome not changed), and the intervention was not used, discuss whether negative results are attributable to lack of uptake and discuss reasons. (Note: Only report in the abstract what the main paper is reporting. If this information is missing from the main body of text, consider adding it)

| | 1 | 2 | 3 | 4 | 5 | |
|---|---|---|---|---|---|---|
| subitem not at all important | ○ | ○ | ○ | ● | ○ | essential |

### Does your paper address subitem 1b-v?

Copy and paste relevant sections from the manuscript abstract (include quotes in quotation marks "like this" to indicate direct quotes from your manuscript), or elaborate on this item by providing additional information not in the ms, or briefly explain why the item is not applicable/relevant for your study

Our results did not have negative results. Like this, " Videoconference assessment of functional status demonstrates higher validity and reliability, as well as higher confidence and satisfaction perceived by patients, than telephone assessment."

## INTRODUCTION

### 2a) In INTRODUCTION: Scientific background and explanation of rationale

### 2a-i) Problem and the type of system/solution

Describe the problem and the type of system/solution that is object of the study: intended as stand-alone intervention vs. incorporated in broader health care program? Intended for a particular patient population? Goals of the intervention, e.g., being more cost-effective to other interventions, replace or complement other solutions? (Note: Details about the intervention are provided in "Methods" under 5)

| | 1 | 2 | 3 | 4 | 5 | |
|---|---|---|---|---|---|---|
| subitem not at all important | ○ | ○ | ○ | ○ | ○ | essential |

Does your paper address subitem 2a-i? *

Copy and paste relevant sections from the manuscript (include quotes in quotation marks "like this" to indicate direct quotes from your manuscript), or elaborate on this item by providing additional information not in the ms, or briefly explain why the item is not applicable/relevant for your study

We described the needs and problems of existing rehabilitation in which clinicians have been difficult to collect post-hospitalization functional data. The solution we proposed is the use of WeChat App, especially videoconference methods to gather the follow-up after the patients discharge from the hospital. We also described this problem has been commonly found in stroke populations. Like this, "Stroke is one of the leading causes of death and disability worldwide [1]. As in other developing countries, the incidence and prevalence of stroke in China are gradually increasing. Each year, China has 2.5 million new stroke cases and more than 11 million stroke survivors; stroke has become the leading cause of death in China [2]. Stroke can have a long-term impact on an individual's physical, mental, and social function, as well as on survivors' caregivers and families [3-6]. The majority of patients receive inpatient stroke rehabilitation to regain function for only the first few weeks after stroke, but functional recovery often occurs 3 months or even longer following a stroke [7]. Additionally, about 30% of post-stroke individuals receive outpatient rehabilitation [8]. Even when offered, access to rehabilitation for patients in China is limited because of transportation, geographical barriers, and monetary factors [9]. Because most patients have recovery potential but do not receive recommended rehabilitation, it is important to develop new strategies to continuously monitor functional recovery and other health outcomes of patients following discharge home in order to better understand the long-term consequences for patients post-stroke [10].
Post-stroke home-based therapies seem to be a viable option for the delivery of stroke care. Follow-up assessments and interventions not only provide a means of monitoring the functional status of patients after transitioning to home and community [11], but also provide instructions to prevent readmission, which is especially important for those who receive a longer stay in inpatient rehabilitation [12]. Moreover, follow-up assessments enable clinicians to adjust the treatment plan for home-based therapies [13,14]. One common method for follow-up assessment is a face-to-face, at-home assessment in which the home health therapist visits the patient at home to perform an evaluation. However, this method demands intensive resources, including the time of trained personnel and financial expenditures [15]. Recent studies have tested alternative methods of follow-up data collection for patients following stroke [16-18]. Telephone administration is a common alternative. This method allows participants to be recruited from diverse geographical areas, is typically less expensive than the face-to-face home assessment, and has a quick turnaround time [19]. Prior studies have found that telephone administration of outcome measures demonstrates equivalent reliability to face-to-face assessment, supporting telephone interview as a feasible solution [20,21]. However, there are some shortcomings to telephone administration. First, many functional measures require a trained therapist to observe and provide ratings of how the patient performs specific daily tasks. Inability to perform observations via telephone administration may be a hindrance to accurately evaluating task performance. Furthermore, telephone administration often assesses survey-based questions, which require patients to have higher education, health literacy, and communication abilities in order to understand the verbal instructions [22]. An earlier study found a large amount of missing data from assessments administered through the telephone interview method for stroke patients and caregivers, limiting the use of this method in clinical trials requiring longitudinal assessments [23].

With advances in computing power and mobile connectivity, many mHealth technologies such as mobile devices, sensors, apps, and social media are becoming available to obtain data pertinent to wellness and disease diagnosis, prevention, and management [24]. WeChat (Chinese version: Weixin), developed in 2011 by Tencent, has become the most common social software app in China [25]. Similar to other social media apps such as Facebook, Twitter, and WhatsApp, WeChat is a free platform that provides seamless opportunities for communication and other mobile applications. People can communicate with one another through the free voice call or video call, as well as instantly share information [26]. According to the Statista Research Department [27], WeChat had over 1.15 billion monthly active users from a wide range of age groups. Harnessing the use of mHealth technologies to improve health and wellness is not uncommon in modern health care [28]. A recent mHealth intervention study utilizing the WeChat app for weight loss behaviors in a group of male workers found promising results: participants who spent more time using the health education program embedded in the WeChat app for engaging in healthy behaviors demonstrated more weight loss [29]. Another mHealth study used the WeChat app to educate parents of pediatric patients undergoing surgery and found that this mobile app–assisted intervention was effective in enhancing parents' knowledge of perioperative procedures [30]. Another study used the WeChat app in a group of discharged patients with head and neck tumors for 6 months and

## 2a-ii) Scientific background, rationale: What is known about the (type of) system

Scientific background, rationale: What is known about the (type of) system that is the object of the study (be sure to discuss the use of similar systems for other conditions/diagnoses, if appropiate), motivation for the study, i.e. what are the reasons for and what is the context for this specific study, from which stakeholder viewpoint is the study performed, potential impact of findings [2]. Briefly justify the choice of the comparator.

| | 1 | 2 | 3 | 4 | 5 | |
|---|---|---|---|---|---|---|
| subitem not at all important | ○ | ○ | ○ | ◉ | ○ | essential |

## Does your paper address subitem 2a-ii? *

Copy and paste relevant sections from the manuscript (include quotes in quotation marks "like this" to indicate direct quotes from your manuscript), or elaborate on this item by providing additional information not in the ms, or briefly explain why the item is not applicable/relevant for your study

Same as above. We described the rationale for using WeChat videoconference as the proposed solution to collect follow-up data. We believed this method can help clinicians get comparable data to the golden standard method, that is face-to-face home assessment but it is less costly method.

## 2b) In INTRODUCTION: Specific objectives or hypotheses

Does your paper address CONSORT subitem 2b? *

Copy and paste relevant sections from the manuscript (include quotes in quotation marks "like this" to indicate direct quotes from your manuscript), or elaborate on this item by providing additional information not in the ms, or briefly explain why the item is not applicable/relevant for your study

This study had two specific aims. The first aim was to compare the validity and reliability of functional assessment between these 2 modes of administration in stroke patients. We hypothesized that videoconference administration would demonstrate higher validity and reliability than telephone administration, because examiners using videoconference administration can observe how the respondent performs specific tasks to provide appropriate ratings, whereas examiners in the telephone administration group demand more subjective appraisals of task performance based on the respondent’s verbal descriptions. Our second aim was to examine the feasibility of the functional assessment administered via videoconference compared to telephone call in stroke patients after rehabilitation hospitalization. We hypothesized that both modes of administration would demonstrate high levels of completion,

## METHODS

## 3a) Description of trial design (such as parallel, factorial) including allocation ratio

Does your paper address CONSORT subitem 3a? *

Copy and paste relevant sections from the manuscript (include quotes in quotation marks "like this" to indicate direct quotes from your manuscript), or elaborate on this item by providing additional information not in the ms, or briefly explain why the item is not applicable/relevant for your study

Like this, "This study was a parallel, 2-group, and pragmatic randomized controlled trial of an mHealth application of functional outcome data collection after inpatient stroke rehabilitation. The trial was registered with the Chinese Clinical Trial Registry (ChiCTR): ChiCTR1900027626. A total of 120 eligible stroke patients from the affiliated rehabilitation hospital of Fujian University of Traditional Chinese Medicine were recruited for this study. Participants were approached by a research assistant, who provided the study information. After participants provided informed consent and were screened for eligibility, participants were randomized into 1 of the 2 WeChat app administration groups: videoconference follow-up or telephone follow-up, with a ratio of 1:1. Eligible participants were randomized using a random number table generated by a study coordinator who was not involved in the recruitment and assessment of participants for the study."

**3b) Important changes to methods after trial commencement (such as eligibility criteria), with reasons**

Does your paper address CONSORT subitem 3b? *

Copy and paste relevant sections from the manuscript (include quotes in quotation marks "like this" to indicate direct quotes from your manuscript), or elaborate on this item by providing additional information not in the ms, or briefly explain why the item is not applicable/relevant for your study

This item is not applicable as we did not changes any methods after trial

3b-i) Bug fixes, Downtimes, Content Changes

Bug fixes, Downtimes, Content Changes: ehealth systems are often dynamic systems. A description of changes to methods therefore also includes important changes made on the intervention or comparator during the trial (e.g., major bug fixes or changes in the functionality or content) (5-iii) and other “unexpected events” that may have influenced study design such as staff changes, system failures/downtimes, etc. [2].

| | 1 | 2 | 3 | 4 | 5 | |
|---|---|---|---|---|---|---|
| subitem not at all important | ○ | ○ | ○ | ● | ○ | essential |

### Does your paper address subitem 3b-i?

Copy and paste relevant sections from the manuscript (include quotes in quotation marks "like this" to indicate direct quotes from your manuscript), or elaborate on this item by providing additional information not in the ms, or briefly explain why the item is not applicable/relevant for your study

This item is not applicable. Fortunately, we did not change the content after the trial commencement. We did not have to fix bugs after the trial commencement

## 4a) Eligibility criteria for participants

### Does your paper address CONSORT subitem 4a? *

Copy and paste relevant sections from the manuscript (include quotes in quotation marks "like this" to indicate direct quotes from your manuscript), or elaborate on this item by providing additional information not in the ms, or briefly explain why the item is not applicable/relevant for your study

Like this, "Patients were eligible to participate in the study if they met the following criteria: (1) aged at least 18 years, (2) diagnosis of first stroke, (3) normal speech function according to the Mandarin Language Screening Test with cut-off scores of >13 for those with primary school education or >14 for those with junior high school or higher education, (4) normal cognitive function according to the Montreal Cognitive Assessment with a cut-off score of >26, and (5) home discharge. Participants were excluded if they (1) did not own a smartphone, (2) were unwilling to install and use the WeChat software on their smartphone, (3) had emotional dysfunction according to the Beck Depression Inventory with a cut-off score of >13, or (4) had other medical illnesses limiting study participation. Because we included only participants who served as their own informant rather than including on a nonselected basis, it is likely that individuals who were too cognitively impaired or were unable to understand the

### 4a-i) Computer / Internet literacy

Computer / Internet literacy is often an implicit “de facto” eligibility criterion - this should be explicitly clarified.

| | 1 | 2 | 3 | 4 | 5 | |
|---|---|---|---|---|---|---|
| subitem not at all important | ○ | ○ | ◉ | ○ | ○ | essential |

### Does your paper address subitem 4a-i?

Copy and paste relevant sections from the manuscript (include quotes in quotation marks "like this" to indicate direct quotes from your manuscript), or elaborate on this item by providing additional information not in the ms, or briefly explain why the item is not applicable/relevant for your study

This item is partly addressed. We recognized that the "computer literacy" might be a potential study bias that this might exclude some potential participants to participate in our study. Thus, in our study criterion, we reported that we only included participants who could served as their own informants. Those who were too cognitively impaired or were unable to understand the study materials and use of the App would be excluded. Like this, "Because we included only participants who served as their own informant rather than including on a non-selected basis, it is likely that individuals who were too cognitively impaired or were unable to understand the study materials were excluded. "

### 4a-ii) Open vs. closed, web-based vs. face-to-face assessments:

Open vs. closed, web-based vs. face-to-face assessments: Mention how participants were recruited (online vs. offline), e.g., from an open access website or from a clinic, and clarify if this was a purely web-based trial, or there were face-to-face components (as part of the intervention or for assessment), i.e., to what degree got the study team to know the participant. In online-only trials, clarify if participants were quasi-anonymous and whether having multiple identities was possible or whether technical or logistical measures (e.g., cookies, email confirmation, phone calls) were used to detect/prevent these.

| | 1 | 2 | 3 | 4 | 5 | |
|---|---|---|---|---|---|---|
| subitem not at all important | ○ | ○ | ○ | ◉ | ○ | essential |

### Does your paper address subitem 4a-ii? *

Copy and paste relevant sections from the manuscript (include quotes in quotation marks "like this" to indicate direct quotes from your manuscript), or elaborate on this item by providing additional information not in the ms, or briefly explain why the item is not applicable/relevant for your study

Like this, "Participants were recruited from the inpatient rehabilitation hospital. Recruitment was initiated while the stroke patient was still in the hospital. The research assistant screened the medical records of all patients undergoing inpatient rehabilitation for stroke. Once potential participants were identified, the research assistant approached the individual and provided information about the study. Participants provided informed consent once they agreed to participate. The research assistant reviewed the inclusion and exclusion criteria and completed the screening tests to confirm eligibility. Participants were then enrolled, and randomization occurred only after recruitment by a study coordinator.

## 4a-iii) Information giving during recruitment

Information given during recruitment. Specify how participants were briefed for recruitment and in the informed consent procedures (e.g., publish the informed consent documentation as appendix, see also item X26), as this information may have an effect on user self-selection, user expectation and may also bias results.

| | 1 | 2 | 3 | 4 | 5 | |
|---|---|---|---|---|---|---|
| subitem not at all important | ○ | ○ | ○ | ● | ○ | essential |

## Does your paper address subitem 4a-iii?

Copy and paste relevant sections from the manuscript (include quotes in quotation marks "like this" to indicate direct quotes from your manuscript), or elaborate on this item by providing additional information not in the ms, or briefly explain why the item is not applicable/relevant for your study

Like this, "Participants were recruited from the inpatient rehabilitation hospital. Recruitment was initiated while the stroke patient was still in the hospital. The research assistant screened the medical records of all patients undergoing inpatient rehabilitation for stroke. Once potential participants were identified, the research assistant approached the individual and provided information about the study. Participants provided informed consent once they agreed to participate. The research assistant reviewed the inclusion and exclusion criteria and completed the screening tests to confirm eligibility. Participants were then enrolled, and randomization occurred only after recruitment by a study coordinator."

## 4b) Settings and locations where the data were collected

### Does your paper address CONSORT subitem 4b? *

Copy and paste relevant sections from the manuscript (include quotes in quotation marks "like this" to indicate direct quotes from your manuscript), or elaborate on this item by providing additional information not in the ms, or briefly explain why the item is not applicable/relevant for your study

We described our study involved the home visit. Like this, "All research assistants received training in assessing the eligibility of potential participants, obtaining informed consent from participants, the study protocol, and obtaining outcome measures for both groups. They also received training in how to coach and assist participants using the WeChat app, including the videoconference and telephone call functions. After randomization, all participants received the baseline assessment at the week of discharge, followed by completion of 2 mHealth application follow-up sessions (either videoconference or telephone), and half of the participants from each group received 2 home visits. The first follow-up session occurred 2 weeks after home discharge. Within 1 week of the first follow-up session, half of the study participants were selected to conduct the first home visit based on stratified sampling in each group. The stratified sampling criteria were grounded on participants' functional abilities. As home visits are costly, this study was limited by randomly selecting half of the study participants for the home visit assessment. The second follow-up session occurred 3 months after home discharge. Within 1 week of the second follow-up session, we completed the second home visit in this subgroup of participants. The time interval between videoconference/telephone follow-up and home visit of 1 week was considered long enough to ensure that the previous responses were forgotten and short enough to ensure that the patient's clinical condition would not substantially change. All assessments were conducted by our trained research assistants with training in physical therapy, occupational therapy, or rehabilitation medicine. To reduce assessor bias in the trial, research assistants who completed the videoconference/telephone follow-up sessions also conducted home visits with patients in the same group. We treated the face-to-face, home visit assessment as the

### 4b-i) Report if outcomes were (self-)assessed through online questionnaires

Clearly report if outcomes were (self-)assessed through online questionnaires (as common in web-based trials) or otherwise.

| | 1 | 2 | 3 | 4 | 5 | |
|---|---|---|---|---|---|---|
| subitem not at all important | ○ | ○ | ◉ | ○ | ○ | essential |

### Does your paper address subitem 4b-i? *

Copy and paste relevant sections from the manuscript (include quotes in quotation marks "like this" to indicate direct quotes from your manuscript), or elaborate on this item by providing additional information not in the ms, or briefly explain why the item is not applicable/relevant for your study

This item is not applicable. We did not collect online questionnaire information.

### 4b-ii) Report how institutional affiliations are displayed

Report how institutional affiliations are displayed to potential participants [on ehealth media], as affiliations with prestigious hospitals or universities may affect volunteer rates, use, and reactions with regards to an intervention.(Not a required item – describe only if this may bias results)

| | 1 | 2 | 3 | 4 | 5 | |
|---|---|---|---|---|---|---|
| subitem not at all important | ○ | ○ | ◉ | ○ | ○ | essential |

### Does your paper address subitem 4b-ii?

Copy and paste relevant sections from the manuscript (include quotes in quotation marks "like this" to indicate direct quotes from your manuscript), or elaborate on this item by providing additional information not in the ms, or briefly explain why the item is not applicable/relevant for your study

This item is not relevant to the study.

## 5) The interventions for each group with sufficient details to allow replication, including how and when they were actually administered

### 5-i) Mention names, credential, affiliations of the developers, sponsors, and owners

Mention names, credential, affiliations of the developers, sponsors, and owners [6] (if authors/evaluators are owners or developer of the software, this needs to be declared in a “Conflict of interest” section or mentioned elsewhere in the manuscript).

| | 1 | 2 | 3 | 4 | 5 | |
|---|---|---|---|---|---|---|
| subitem not at all important | ○ | ○ | ○ | ◉ | ○ | essential |

## Does your paper address subitem 5-i?

Copy and paste relevant sections from the manuscript (include quotes in quotation marks "like this" to indicate direct quotes from your manuscript), or elaborate on this item by providing additional information not in the ms, or briefly explain why the item is not applicable/relevant for your study

We described detailed information about the App use and developers.

## 5-ii) Describe the history/development process

Describe the history/development process of the application and previous formative evaluations (e.g., focus groups, usability testing), as these will have an impact on adoption/use rates and help with interpreting results.

| | 1 | 2 | 3 | 4 | 5 | |
|---|---|---|---|---|---|---|
| subitem not at all important | ○ | ○ | ● | ○ | ○ | essential |

## Does your paper address subitem 5-ii?

Copy and paste relevant sections from the manuscript (include quotes in quotation marks "like this" to indicate direct quotes from your manuscript), or elaborate on this item by providing additional information not in the ms, or briefly explain why the item is not applicable/relevant for your study

This item is not applicable to the study. The App used in the study was not originally developed by our research team. We used the "off-the-shelf" method to choose this App. This is a publicly download App.

## 5-iii) Revisions and updating

Revisions and updating. Clearly mention the date and/or version number of the application/intervention (and comparator, if applicable) evaluated, or describe whether the intervention underwent major changes during the evaluation process, or whether the development and/or content was “frozen” during the trial. Describe dynamic components such as news feeds or changing content which may have an impact on the replicability of the intervention (for unexpected events see item 3b).

| | 1 | 2 | 3 | 4 | 5 | |
|---|---|---|---|---|---|---|
| subitem not at all important | ○ | ○ | ○ | ● | ○ | essential |

### Does your paper address subitem 5-iii?

Copy and paste relevant sections from the manuscript (include quotes in quotation marks "like this" to indicate direct quotes from your manuscript), or elaborate on this item by providing additional information not in the ms, or briefly explain why the item is not applicable/relevant for your study

This item is not applicable to the study. The App used in the study was not originally developed by our research team.

### 5-iv) Quality assurance methods

Provide information on quality assurance methods to ensure accuracy and quality of information provided [1], if applicable.

| | 1 | 2 | 3 | 4 | 5 | |
|---|---|---|---|---|---|---|
| subitem not at all important | ○ | ○ | ◉ | ○ | ○ | essential |

### Does your paper address subitem 5-iv?

Copy and paste relevant sections from the manuscript (include quotes in quotation marks "like this" to indicate direct quotes from your manuscript), or elaborate on this item by providing additional information not in the ms, or briefly explain why the item is not applicable/relevant for your study

We provided formal training to our research assistants to collect the data to ensure high accuracy and quality. Like this, "All research assistants received training in assessing the eligibility of potential participants, obtaining informed consent from participants, the study protocol, and obtaining outcome measures for both groups. They also received training in how to coach and assist participants using the WeChat app, including the videoconference and telephone call functions. "

### 5-v) Ensure replicability by publishing the source code, and/or providing screenshots/screen-capture video, and/or providing flowcharts of the algorithms used

Ensure replicability by publishing the source code, and/or providing screenshots/screen-capture video, and/or providing flowcharts of the algorithms used. Replicability (i.e., other researchers should in principle be able to replicate the study) is a hallmark of scientific reporting.

| | 1 | 2 | 3 | 4 | 5 | |
|---|---|---|---|---|---|---|
| subitem not at all important | ○ | ○ | ○ | ◉ | ○ | essential |

## Does your paper address subitem 5-v?

Copy and paste relevant sections from the manuscript (include quotes in quotation marks "like this" to indicate direct quotes from your manuscript), or elaborate on this item by providing additional information not in the ms, or briefly explain why the item is not applicable/relevant for your study

See Figure 1 in the manuscript.

## 5-vi) Digital preservation

Digital preservation: Provide the URL of the application, but as the intervention is likely to change or disappear over the course of the years; also make sure the intervention is archived (Internet Archive, webcitation.org, and/or publishing the source code or screenshots/videos alongside the article). As pages behind login screens cannot be archived, consider creating demo pages which are accessible without login.

| | 1 | 2 | 3 | 4 | 5 | |
|---|---|---|---|---|---|---|
| subitem not at all important | ○ | ○ | ◉ | ○ | ○ | essential |

## Does your paper address subitem 5-vi?

Copy and paste relevant sections from the manuscript (include quotes in quotation marks "like this" to indicate direct quotes from your manuscript), or elaborate on this item by providing additional information not in the ms, or briefly explain why the item is not applicable/relevant for your study

This item is not applicable to the study. The App used in the study was not originally developed by our research team.

## 5-vii) Access

Access: Describe how participants accessed the application, in what setting/context, if they had to pay (or were paid) or not, whether they had to be a member of specific group. If known, describe how participants obtained “access to the platform and Internet” [1]. To ensure access for editors/reviewers/readers, consider to provide a “backdoor” login account or demo mode for reviewers/readers to explore the application (also important for archiving purposes, see vi).

| | 1 | 2 | 3 | 4 | 5 | |
|---|---|---|---|---|---|---|
| subitem not at all important | ○ | ○ | ○ | ◉ | ○ | essential |

Does your paper address subitem 5-vii? *

Copy and paste relevant sections from the manuscript (include quotes in quotation marks "like this" to indicate direct quotes from your manuscript), or elaborate on this item by providing additional information not in the ms, or briefly explain why the item is not applicable/relevant for your study

This App is a publicly available and free App. Thus, participants did not have to pay or have other restrictions to access this App. We excluded the participants who were unwilling to install and use the software on their smartphone. Like this, "Participants were excluded if they (1) did not own a smartphone, (2) were unwilling to install and use the WeChat software on their smartphone"

5-viii) Mode of delivery, features/functionalities/components of the intervention and comparator, and the theoretical framework

Describe mode of delivery, features/functionalities/components of the intervention and comparator, and the theoretical framework [6] used to design them (instructional strategy [1], behaviour change techniques, persuasive features, etc., see e.g., [7, 8] for terminology). This includes an in-depth description of the content (including where it is coming from and who developed it) [1],” whether [and how] it is tailored to individual circumstances and allows users to track their progress and receive feedback” [6]. This also includes a description of communication delivery channels and – if computer-mediated communication is a component – whether communication was synchronous or asynchronous [6]. It also includes information on presentation strategies [1], including page design principles, average amount of text on pages, presence of hyperlinks to other resources, etc. [1].

| | 1 | 2 | 3 | 4 | 5 | |
|---|---|---|---|---|---|---|
| subitem not at all important | ○ | ○ | ○ | ● | ○ | essential |

Does your paper address subitem 5-viii? *

Copy and paste relevant sections from the manuscript (include quotes in quotation marks "like this" to indicate direct quotes from your manuscript), or elaborate on this item by providing additional information not in the ms, or briefly explain why the item is not applicable/relevant for your study

We described details about different intervention arms, and their procedures. Like this, "All research assistants received training in assessing the eligibility of potential participants, obtaining informed consent from participants, the study protocol, and obtaining outcome measures for both groups. They also received training in how to coach and assist participants using the WeChat app, including the videoconference and telephone call functions. After randomization, all participants received the baseline assessment at the week of discharge, followed by completion of 2 mHealth application follow-up sessions (either videoconference or telephone), and half of the participants from each group received 2 home visits. The first follow-up session occurred 2 weeks after home discharge. Within 1 week of the first follow-up session, half of the study participants were selected to conduct the first home visit based on stratified sampling in each group. The stratified sampling criteria were grounded on participants' functional abilities. As home visits are costly, this study was limited by randomly selecting half of the study participants for the home visit assessment. The second follow-up session occurred 3 months after home discharge. Within 1 week of the second follow-up session, we completed the second home visit in this subgroup of participants. The time interval between videoconference/telephone follow-up and home visit of 1 week was considered long enough to ensure that the previous responses were forgotten and short enough to ensure that the patient's clinical condition would not substantially change. All assessments were conducted by our trained research assistants with training in physical therapy, occupational therapy, or rehabilitation medicine. To reduce assessor bias in the trial, research assistants who completed the videoconference/telephone follow-up sessions also conducted home visits with patients in the same group. We treated the face-to-face, home visit assessment as the gold standard in this study.
Participants in the videoconference follow-up group received training on the usage of the videoconference function of the WeChat app before discharge. During the follow-up sessions, research assistants asked participants to complete individual functional tasks and rate their actual performance through the videoconference, with the exception of bladder and bowel management tasks, which were assessed by the participant's verbal descriptions. Participants also described difficulties pertaining to their individual task performance. During the home visit, research assistants completed the face-to-face observations by rating participants as they completed the same functional tasks. We used the same scoring criteria to evaluate the performance of our study participants in both videoconference and face-to-face, home visit assessments. Participants in the telephone follow-up group received training on the usage of the telephone function of the WeChat app before discharge. During the follow-up sessions, research assistants made telephone calls, asked participants how they performed in each functional item, and appraised their performance based on the participant's verbal descriptions. During the home visit, research assistants completed the same protocol as in the videoconference follow-up group. We used the same

## 5-ix) Describe use parameters

Describe use parameters (e.g., intended “doses” and optimal timing for use). Clarify what instructions or recommendations were given to the user, e.g., regarding timing, frequency, heaviness of use, if any, or was the intervention used ad libitum.

| | 1 | 2 | 3 | 4 | 5 | |
|---|---|---|---|---|---|---|
| subitem not at all important | ○ | ○ | ○ | ◉ | ○ | essential |

## Does your paper address subitem 5-ix?

Copy and paste relevant sections from the manuscript (include quotes in quotation marks "like this" to indicate direct quotes from your manuscript), or elaborate on this item by providing additional information not in the ms, or briefly explain why the item is not applicable/relevant for your study

Same as above response 5-viii

## 5-x) Clarify the level of human involvement

Clarify the level of human involvement (care providers or health professionals, also technical assistance) in the e-intervention or as co-intervention (detail number and expertise of professionals involved, if any, as well as “type of assistance offered, the timing and frequency of the support, how it is initiated, and the medium by which the assistance is delivered”. It may be necessary to distinguish between the level of human involvement required for the trial, and the level of human involvement required for a routine application outside of a RCT setting (discuss under item 21 – generalizability).

| | 1 | 2 | 3 | 4 | 5 | |
|---|---|---|---|---|---|---|
| subitem not at all important | ○ | ○ | ○ | ◉ | ○ | essential |

## Does your paper address subitem 5-x?

Copy and paste relevant sections from the manuscript (include quotes in quotation marks "like this" to indicate direct quotes from your manuscript), or elaborate on this item by providing additional information not in the ms, or briefly explain why the item is not applicable/relevant for your study

Same as above response 5-viii

## 5-xi) Report any prompts/reminders used

Report any prompts/reminders used: Clarify if there were prompts (letters, emails, phone calls, SMS) to use the application, what triggered them, frequency etc. It may be necessary to distinguish between the level of prompts/reminders required for the trial, and the level of prompts/reminders for a routine application outside of a RCT setting (discuss under item 21 – generalizability).

| | 1 | 2 | 3 | 4 | 5 | |
|---|---|---|---|---|---|---|
| subitem not at all important | ○ | ○ | ◉ | ○ | ○ | essential |

## Does your paper address subitem 5-xi? *

Copy and paste relevant sections from the manuscript (include quotes in quotation marks "like this" to indicate direct quotes from your manuscript), or elaborate on this item by providing additional information not in the ms, or briefly explain why the item is not applicable/relevant for your study

This item is not applicable.

## 5-xii) Describe any co-interventions (incl. training/support)

Describe any co-interventions (incl. training/support): Clearly state any interventions that are provided in addition to the targeted eHealth intervention, as ehealth intervention may not be designed as stand-alone intervention. This includes training sessions and support [1]. It may be necessary to distinguish between the level of training required for the trial, and the level of training for a routine application outside of a RCT setting (discuss under item 21 – generalizability.

| | 1 | 2 | 3 | 4 | 5 | |
|---|---|---|---|---|---|---|
| subitem not at all important | ○ | ○ | ○ | ◉ | ○ | essential |

### Does your paper address subitem 5-xii? *

Copy and paste relevant sections from the manuscript (include quotes in quotation marks "like this" to indicate direct quotes from your manuscript), or elaborate on this item by providing additional information not in the ms, or briefly explain why the item is not applicable/relevant for your study

We described details about different intervention arms, and their procedures. Like this, "All research assistants received training in assessing the eligibility of potential participants, obtaining informed consent from participants, the study protocol, and obtaining outcome measures for both groups. They also received training in how to coach and assist participants using the WeChat app, including the videoconference and telephone call functions. After randomization, all participants received the baseline assessment at the week of discharge, followed by completion of 2 mHealth application follow-up sessions (either videoconference or telephone), and half of the participants from each group received 2 home visits. The first follow-up session occurred 2 weeks after home discharge. Within 1 week of the first follow-up session, half of the study participants were selected to conduct the first home visit based on stratified sampling in each group. The stratified sampling criteria were grounded on participants' functional abilities. As home visits are costly, this study was limited by randomly selecting half of the study participants for the home visit assessment. The second follow-up session occurred 3 months after home discharge. Within 1 week of the second follow-up session, we completed the second home visit in this subgroup of participants. The time interval between videoconference/telephone follow-up and home visit of 1 week was considered long enough to ensure that the previous responses were forgotten and short enough to ensure that the patient's clinical condition would not substantially change. All assessments were conducted by our trained research assistants with training in physical therapy, occupational therapy, or rehabilitation medicine. To reduce assessor bias in the trial, research assistants who completed the videoconference/telephone follow-up sessions also conducted home visits with patients in the same group. We treated the face-to-face, home visit assessment as the gold standard in this study.

Participants in the videoconference follow-up group received training on the usage of the videoconference function of the WeChat app before discharge. During the follow-up sessions, research assistants asked participants to complete individual functional tasks and rate their actual performance through the videoconference, with the exception of bladder and bowel management tasks, which were assessed by the participant's verbal descriptions. Participants also described difficulties pertaining to their individual task performance. During the home visit, research assistants completed the face-to-face observations by rating participants as they completed the same functional tasks. We used the same scoring criteria to evaluate the performance of our study participants in both videoconference and face-to-face, home visit assessments. Participants in the telephone follow-up group received training on the usage of the telephone function of the WeChat app before discharge. During the follow-up sessions, research assistants made telephone calls, asked participants how they performed in each functional item, and appraised their performance based on the participant's verbal descriptions. During the home visit, research assistants completed the same protocol as in the videoconference follow-up group. We used the same

## 6a) Completely defined pre-specified primary and secondary outcome measures, including how and when they were assessed

Does your paper address CONSORT subitem 6a? *

Copy and paste relevant sections from the manuscript (include quotes in quotation marks "like this" to indicate direct quotes from your manuscript), or elaborate on this item by providing additional information not in the ms, or briefly explain why the item is not applicable/relevant for your study

Like this, "As recommended in a systematic review of optimal outcome measures for stroke therapy trials [32], the primary outcome selected for this study was improvement in activities and participation rather than the reduction of impairments. Thus, we chose functional status (ie, the performance of activities of daily living [ADLs]) of stroke participants as our primary outcome variable. We used the Modified Barthel Index (MBI) to assess the performance of ADLs in the 2 groups [33,34]. The MBI can be administered using clinician-rated or patient-reported methods. It includes 10 items measuring grooming, bathing, feeding, toileting, stair climbing, dressing, bowel control, bladder control, mobility, and chair/bed transfer. Items have different response options, with anchored scores provided for different options. The total score ranges from 0–100. A higher score means that the participant has greater independence. Adequate validity and reliability were found for the Chinese version of the MBI used in this study [35].
We also defined 2 outcome variables to examine the feasibility of using an mHealth application to measure functional status of stroke participants in this study. The first variable was the completion of the MBI among our study participants in both groups at both follow-up periods. The second variable was the acceptability among our study participants (ie, levels of satisfaction, comfort, and confidence) of using videoconference/telephone to complete the functional assessment at follow-up periods. We developed 3 questions: (1) “Are you satisfied with this follow-up assessment?” (2) “Are you comfortable with this follow-up assessment?” and (3) “Are you confident using this follow-up assessment?” All items were rated on a 4-point

## 6a-i) Online questionnaires: describe if they were validated for online use and apply CHERRIES items to describe how the questionnaires were designed/deployed

If outcomes were obtained through online questionnaires, describe if they were validated for online use and apply CHERRIES items to describe how the questionnaires were designed/deployed [9].

| | 1 | 2 | 3 | 4 | 5 | |
|---|---|---|---|---|---|---|
| subitem not at all important | ○ | ○ | ● | ○ | ○ | essential |

Does your paper address subitem 6a-i?

Copy and paste relevant sections from manuscript text

This item is not relevant to the study. We did not use any online questionnaire.

6a-ii) Describe whether and how "use" (including intensity of use/dosage) was defined/measured/monitored

Describe whether and how "use" (including intensity of use/dosage) was defined/measured/monitored (logins, logfile analysis, etc.). Use/adoption metrics are important process outcomes that should be reported in any ehealth trial.

| | 1 | 2 | 3 | 4 | 5 | |
|---|---|---|---|---|---|---|
| subitem not at all important | ○ | ○ | ○ | ◉ | ○ | essential |

Does your paper address subitem 6a-ii?

Copy and paste relevant sections from manuscript text

We reported the completion rate of our outcome assessment to indicate the acceptability/adoption of different modes of assessment administration. Like this "The first variable was the completion of the MBI among our study participants in both groups at both follow-up periods"

6a-iii) Describe whether, how, and when qualitative feedback from participants was obtained

Describe whether, how, and when qualitative feedback from participants was obtained (e.g., through emails, feedback forms, interviews, focus groups).

| | 1 | 2 | 3 | 4 | 5 | |
|---|---|---|---|---|---|---|
| subitem not at all important | ○ | ○ | ○ | ◉ | ○ | essential |

Does your paper address subitem 6a-iii?

Copy and paste relevant sections from manuscript text

We reported that we collected the acceptability about their satisfction, comfort and confidence of using App for the study assessment. Like this, "The second variable was the acceptability among our study participants (ie, levels of satisfaction, comfort, and confidence) of using videoconference/telephone to complete the functional assessment at follow-up periods. We developed 3 questions: (1) “Are you satisfied with this follow-up assessment?” (2) “Are you comfortable with this follow-up assessment?” and (3) “Are you confident using this follow-up assessment?” All items were rated on a 4-point scale ranging from “very satisfied/comfortable/confident” to “unsatisfied/uncomfortable/unconfident.”"

## 6b) Any changes to trial outcomes after the trial commenced, with reasons

Does your paper address CONSORT subitem 6b? *

Copy and paste relevant sections from the manuscript (include quotes in quotation marks "like this" to indicate direct quotes from your manuscript), or elaborate on this item by providing additional information not in the ms, or briefly explain why the item is not applicable/relevant for your study

This item is not applicable. We did not change any trial outcomes.

## 7a) How sample size was determined

NPT: When applicable, details of whether and how the clustering by care provides or centers was addressed

7a-i) Describe whether and how expected attrition was taken into account when calculating the sample size

Describe whether and how expected attrition was taken into account when calculating the sample size.

| | 1 | 2 | 3 | 4 | 5 | |
|---|---|---|---|---|---|---|
| subitem not at all important | ○ | ○ | ◉ | ○ | ○ | essential |

Does your paper address subitem 7a-i?

Copy and paste relevant sections from manuscript title (include quotes in quotation marks "like this" to indicate direct quotes from your manuscript), or elaborate on this item by providing additional information not in the ms, or briefly explain why the item is not applicable/relevant for your study

We did not compute the power prior to conducting the study.

## 7b) When applicable, explanation of any interim analyses and stopping guidelines

Does your paper address CONSORT subitem 7b? *

Copy and paste relevant sections from the manuscript (include quotes in quotation marks "like this" to indicate direct quotes from your manuscript), or elaborate on this item by providing additional information not in the ms, or briefly explain why the item is not applicable/relevant for your study

This item is not applicable.

## 8a) Method used to generate the random allocation sequence

NPT: When applicable, how care providers were allocated to each trial group

Does your paper address CONSORT subitem 8a? *

Copy and paste relevant sections from the manuscript (include quotes in quotation marks "like this" to indicate direct quotes from your manuscript), or elaborate on this item by providing additional information not in the ms, or briefly explain why the item is not applicable/relevant for your study

Like this, "Eligible participants were randomized using a random number table generated by a study coordinator who was not involved in the recruitment and assessment of participants for the study."

## 8b) Type of randomisation; details of any restriction (such as blocking and block size)

Does your paper address CONSORT subitem 8b? *

Copy and paste relevant sections from the manuscript (include quotes in quotation marks "like this" to indicate direct quotes from your manuscript), or elaborate on this item by providing additional information not in the ms, or briefly explain why the item is not applicable/relevant for your study

Eligible participants were randomized using simple randomization with random number generation. SAS random number generator was used to ensure that the final sample was uniformly distributed . The research coordinator was responsible for

## 9) Mechanism used to implement the random allocation sequence (such as sequentially numbered containers), describing any steps taken to conceal the sequence until interventions were assigned

Does your paper address CONSORT subitem 9? *

Copy and paste relevant sections from the manuscript (include quotes in quotation marks "like this" to indicate direct quotes from your manuscript), or elaborate on this item by providing additional information not in the ms, or briefly explain why the item is not applicable/relevant for your study

See above response 8b.

## 10) Who generated the random allocation sequence, who enrolled participants, and who assigned participants to interventions

Does your paper address CONSORT subitem 10? *

Copy and paste relevant sections from the manuscript (include quotes in quotation marks "like this" to indicate direct quotes from your manuscript), or elaborate on this item by providing additional information not in the ms, or briefly explain why the item is not applicable/relevant for your study

See above response 8b.

## 11a) If done, who was blinded after assignment to interventions (for example, participants, care providers, those assessing outcomes) and how

NPT: Whether or not administering co-interventions were blinded to group assignment

## 11a-i) Specify who was blinded, and who wasn’t

Specify who was blinded, and who wasn’t. Usually, in web-based trials it is not possible to blind the participants [1, 3] (this should be clearly acknowledged), but it may be possible to blind outcome assessors, those doing data analysis or those administering co-interventions (if any).

| | 1 | 2 | 3 | 4 | 5 | |
|---|---|---|---|---|---|---|
| subitem not at all important | ○ | ○ | ○ | ◉ | ○ | essential |

## Does your paper address subitem 11a-i? *

Copy and paste relevant sections from the manuscript (include quotes in quotation marks "like this" to indicate direct quotes from your manuscript), or elaborate on this item by providing additional information not in the ms, or briefly explain why the item is not applicable/relevant for your study

We designated a research coordinator who was not involved in the recruitment and assessment of participants for the study for generating this random number.

## 11a-ii) Discuss e.g., whether participants knew which intervention was the “intervention of interest” and which one was the “comparator”

Informed consent procedures (4a-ii) can create biases and certain expectations - discuss e.g., whether participants knew which intervention was the “intervention of interest” and which one was the “comparator”.

| | 1 | 2 | 3 | 4 | 5 | |
|---|---|---|---|---|---|---|
| subitem not at all important | ○ | ○ | ○ | ◉ | ○ | essential |

## Does your paper address subitem 11a-ii?

Copy and paste relevant sections from the manuscript (include quotes in quotation marks "like this" to indicate direct quotes from your manuscript), or elaborate on this item by providing additional information not in the ms, or briefly explain why the item is not applicable/relevant for your study

We randomized the participants into two groups using simple randomization. Besides, we did not inform the participants which treatment arm was the intervention of interest, which arm was the comparator.

### 11b) If relevant, description of the similarity of interventions

(this item is usually not relevant for ehealth trials as it refers to similarity of a placebo or sham intervention to a active medication/intervention)

Does your paper address CONSORT subitem 11b? *

Copy and paste relevant sections from the manuscript (include quotes in quotation marks "like this" to indicate direct quotes from your manuscript), or elaborate on this item by providing additional information not in the ms, or briefly explain why the item is not applicable/relevant for your study

This item is not applicable to the study.

### 12a) Statistical methods used to compare groups for primary and secondary outcomes

NPT: When applicable, details of whether and how the clustering by care providers or centers was addressed

Does your paper address CONSORT subitem 12a? *

Copy and paste relevant sections from the manuscript (include quotes in quotation marks "like this" to indicate direct quotes from your manuscript), or elaborate on this item by providing additional information not in the ms, or briefly explain why the item is not applicable/relevant for your study

Like this, "Data were analyzed using the Statistical Package for Social Sciences, version 20 (IBM, Chicago, IL, USA). Baseline characteristics between groups were compared using t tests or Mann-Whitney tests for continuous variables, and Pearson's chi-square tests for categorical variables. We applied Wilcoxon matched-pairs signed-rank tests to compare MBI score differences between videoconference and telephone assessments, as well as between videoconference/telephone and home visit assessments for the validity evaluation. We set the P value to 0.05 for statistical significance. We used intraclass correlation coefficients (ICCs) to evaluate agreement of all item scores between these assessments for the reliability evaluation. According to Landis and Koch [36], the ICC can theoretically vary between 0 and 1.0, where an ICC of 0 indicates no reliability, and an ICC of 1.0 indicates perfect reliability; ICCs above .80 indicate acceptable reliability. We used chi-square tests or Fisher's exact tests to compare rates of completion, satisfaction, comfort, and confidence between the videoconference and telephone modes of administration."

## 12a-i) Imputation techniques to deal with attrition / missing values

Imputation techniques to deal with attrition / missing values: Not all participants will use the intervention/comparator as intended and attrition is typically high in ehealth trials. Specify how participants who did not use the application or dropped out from the trial were treated in the statistical analysis (a complete case analysis is strongly discouraged, and simple imputation techniques such as LOCF may also be problematic [4]).

| | 1 | 2 | 3 | 4 | 5 | |
|---|---|---|---|---|---|---|
| subitem not at all important | ○ | ○ | ○ | ◉ | ○ | essential |

## Does your paper address subitem 12a-i? *

Copy and paste relevant sections from the manuscript (include quotes in quotation marks "like this" to indicate direct quotes from your manuscript), or elaborate on this item by providing additional information not in the ms, or briefly explain why the item is not applicable/relevant for your study

We did not impute any missing data for the analyses.

## 12b) Methods for additional analyses, such as subgroup analyses and adjusted analyses

## Does your paper address CONSORT subitem 12b? *

Copy and paste relevant sections from the manuscript (include quotes in quotation marks "like this" to indicate direct quotes from your manuscript), or elaborate on this item by providing additional information not in the ms, or briefly explain why the item is not applicable/relevant for your study

We did not perform subgroup analyses in the study.

## X26) REB/IRB Approval and Ethical Considerations [recommended as subheading under "Methods"] (not a CONSORT item)

## X26-i) Comment on ethics committee approval

| | 1 | 2 | 3 | 4 | 5 | |
|---|---|---|---|---|---|---|
| subitem not at all important | ○ | ○ | ○ | ○ | ◉ | essential |

## Does your paper address subitem X26-i?

Copy and paste relevant sections from the manuscript (include quotes in quotation marks "like this" to indicate direct quotes from your manuscript), or elaborate on this item by providing additional information not in the ms, or briefly explain why the item is not applicable/relevant for your study

Like this, "This trial was implemented in compliance with the declaration of Helsinki and approved by the Ethics Board of the affiliated rehabilitation hospital of FJTCM (number: 2016KY-032-01). All participants provided informed, written consent before participation. Participants received an honorarium to acknowledge their research

## x26-ii) Outline informed consent procedures

Outline informed consent procedures e.g., if consent was obtained offline or online (how? Checkbox, etc.?), and what information was provided (see 4a-ii). See [6] for some items to be included in informed consent documents.

| | 1 | 2 | 3 | 4 | 5 | |
|---|---|---|---|---|---|---|
| subitem not at all important | ○ | ○ | ○ | ○ | ◉ | essential |

### Does your paper address subitem X26-ii?

Copy and paste relevant sections from the manuscript (include quotes in quotation marks "like this" to indicate direct quotes from your manuscript), or elaborate on this item by providing additional information not in the ms, or briefly explain why the item is not applicable/relevant for your study

Like this "Participants were recruited from the inpatient rehabilitation hospital. Recruitment was initiated while the stroke patient was still in the hospital. The research assistant screened the medical records of all patients undergoing inpatient rehabilitation for stroke. Once potential participants were identified, the research assistant approached the individual and provided information about the study. Participants provided informed consent once they agreed to participate. The research assistant reviewed the inclusion and exclusion criteria and completed the screening tests to confirm eligibility. Participants were then enrolled, and randomization occurred only after recruitment by a study coordinator."

### X26-iii) Safety and security procedures

Safety and security procedures, incl. privacy considerations, and any steps taken to reduce the likelihood or detection of harm (e.g., education and training, availability of a hotline)

| | 1 | 2 | 3 | 4 | 5 | |
|---|---|---|---|---|---|---|
| subitem not at all important | ○ | ○ | ○ | ● | ○ | essential |

### Does your paper address subitem X26-iii?

Copy and paste relevant sections from the manuscript (include quotes in quotation marks "like this" to indicate direct quotes from your manuscript), or elaborate on this item by providing additional information not in the ms, or briefly explain why the item is not applicable/relevant for your study

We provided training to our research assistants to conduct the standardized assessments. Like this "All research assistants received training in assessing the eligibility of potential participants, obtaining informed consent from participants, the study protocol, and obtaining outcome measures for both groups. They also received training in how to coach and assist participants using the WeChat app, including the videoconference and telephone call functions. "

## RESULTS

## 13a) For each group, the numbers of participants who were randomly assigned, received intended treatment, and were analysed for the primary outcome

NPT: The number of care providers or centers performing the intervention in each group and the number of patients treated by each care provider in each center

Does your paper address CONSORT subitem 13a? *

Copy and paste relevant sections from the manuscript (include quotes in quotation marks "like this" to indicate direct quotes from your manuscript), or elaborate on this item by providing additional information not in the ms, or briefly explain why the item is not applicable/relevant for your study

See Table 1 of the manuscript.

## 13b) For each group, losses and exclusions after randomisation, together with reasons

Does your paper address CONSORT subitem 13b? (NOTE: Preferably, this is shown in a CONSORT flow diagram) *

Copy and paste relevant sections from the manuscript (include quotes in quotation marks "like this" to indicate direct quotes from your manuscript), or elaborate on this item by providing additional information not in the ms, or briefly explain why the item is not applicable/relevant for your study

See Figure 1 for reasons to loss .

13b-i) Attrition diagram

Strongly recommended: An attrition diagram (e.g., proportion of participants still logging in or using the intervention/comparator in each group plotted over time, similar to a survival curve) or other figures or tables demonstrating usage/dose/engagement.

| | 1 | 2 | 3 | 4 | 5 | |
|---|---|---|---|---|---|---|
| subitem not at all important | ○ | ○ | ○ | ◉ | ○ | essential |

### Does your paper address subitem 13b-i?

Copy and paste relevant sections from the manuscript or cite the figure number if applicable (include quotes in quotation marks "like this" to indicate direct quotes from your manuscript), or elaborate on this item by providing additional information not in the ms, or briefly explain why the item is not applicable/relevant for your study

See Figure 1

## 14a) Dates defining the periods of recruitment and follow-up

### Does your paper address CONSORT subitem 14a? *

Copy and paste relevant sections from the manuscript (include quotes in quotation marks "like this" to indicate direct quotes from your manuscript), or elaborate on this item by providing additional information not in the ms, or briefly explain why the item is not applicable/relevant for your study

Like this, "The first follow-up session occurred 2 weeks after home discharge. Within 1 week of the first follow-up session, half of the study participants were selected to conduct the first home visit based on stratified sampling in each group. The stratified sampling criteria were grounded on participants' functional abilities. As home visits are costly, this study was limited by randomly selecting half of the study participants for the home visit assessment. The second follow-up session occurred 3 months after home discharge. Within 1 week of the second follow-up session, we completed the second home visit in this subgroup of participants. The time interval between videoconference/telephone follow-up and home visit of 1 week was considered long enough to ensure that the previous responses were forgotten and short enough to ensure that the patient's clinical condition would not substantially change. All assessments were conducted by our trained research assistants with training in physical therapy, occupational therapy, or rehabilitation medicine. "

### 14a-i) Indicate if critical "secular events" fell into the study period

Indicate if critical "secular events" fell into the study period, e.g., significant changes in Internet resources available or "changes in computer hardware or Internet delivery resources"

| | 1 | 2 | 3 | 4 | 5 | |
|---|---|---|---|---|---|---|
| subitem not at all important | ○ | ○ | ● | ○ | ○ | essential |

### Does your paper address subitem 14a-i?

Copy and paste relevant sections from the manuscript (include quotes in quotation marks "like this" to indicate direct quotes from your manuscript), or elaborate on this item by providing additional information not in the ms, or briefly explain why the item is not applicable/relevant for your study

This item is not applicable.

## 14b) Why the trial ended or was stopped (early)

### Does your paper address CONSORT subitem 14b? *

Copy and paste relevant sections from the manuscript (include quotes in quotation marks "like this" to indicate direct quotes from your manuscript), or elaborate on this item by providing additional information not in the ms, or briefly explain why the item is not applicable/relevant for your study

No.

## 15) A table showing baseline demographic and clinical characteristics for each group

NPT: When applicable, a description of care providers (case volume, qualification, expertise, etc.) and centers (volume) in each group

### Does your paper address CONSORT subitem 15? *

Copy and paste relevant sections from the manuscript (include quotes in quotation marks "like this" to indicate direct quotes from your manuscript), or elaborate on this item by providing additional information not in the ms, or briefly explain why the item is not applicable/relevant for your study

Table 1

15-i) Report demographics associated with digital divide issues

In ehealth trials it is particularly important to report demographics associated with digital divide issues, such as age, education, gender, social-economic status, computer/Internet/ehealth literacy of the participants, if known.

| | 1 | 2 | 3 | 4 | 5 | |
|---|---|---|---|---|---|---|
| subitem not at all important | ○ | ○ | ○ | ◉ | ○ | essential |

Does your paper address subitem 15-i? *

Copy and paste relevant sections from the manuscript (include quotes in quotation marks "like this" to indicate direct quotes from your manuscript), or elaborate on this item by providing additional information not in the ms, or briefly explain why the item is not applicable/relevant for your study

We reported demographic information, including age, gender, education and, occupation in each of two groups but we did not collect computer literacy. See Table 1

**16) For each group, number of participants (denominator) included in each analysis and whether the analysis was by original assigned groups**

16-i) Report multiple “denominators” and provide definitions

Report multiple “denominators” and provide definitions: Report N’s (and effect sizes) “across a range of study participation [and use] thresholds” [1], e.g., N exposed, N consented, N used more than x times, N used more than y weeks, N participants “used” the intervention/comparator at specific pre-defined time points of interest (in absolute and relative numbers per group). Always clearly define “use” of the intervention.

| | 1 | 2 | 3 | 4 | 5 | |
|---|---|---|---|---|---|---|
| subitem not at all important | ○ | ○ | ○ | ◉ | ○ | essential |

Does your paper address subitem 16-i? *

Copy and paste relevant sections from the manuscript (include quotes in quotation marks "like this" to indicate direct quotes from your manuscript), or elaborate on this item by providing additional information not in the ms, or briefly explain why the item is not applicable/relevant for your study

We reported this information accordingly. Like this, "Of the initial 60 participants in each group, 49 participants (82%) in the videoconference follow-up group and 48 participants (80%) in the telephone follow-up group completed the entire study protocol. " Also, please see Table 1 of the manuscript.

16-ii) Primary analysis should be intent-to-treat

Primary analysis should be intent-to-treat, secondary analyses could include comparing only "users", with the appropriate caveats that this is no longer a randomized sample (see 18-i).

| | 1 | 2 | 3 | 4 | 5 | |
|---|---|---|---|---|---|---|
| subitem not at all important | ○ | ○ | ◉ | ○ | ○ | essential |

Does your paper address subitem 16-ii?

Copy and paste relevant sections from the manuscript (include quotes in quotation marks "like this" to indicate direct quotes from your manuscript), or elaborate on this item by providing additional information not in the ms, or briefly explain why the item is not applicable/relevant for your study

We included all 60 participants in each group for the primary analysis.

**17a) For each primary and secondary outcome, results for each group, and the estimated effect size and its precision (such as 95% confidence interval)**

Does your paper address CONSORT subitem 17a? *

Copy and paste relevant sections from the manuscript (include quotes in quotation marks "like this" to indicate direct quotes from your manuscript), or elaborate on this item by providing additional information not in the ms, or briefly explain why the item is not applicable/relevant for your study

See Table 5 and Table 6

## 17a-i) Presentation of process outcomes such as metrics of use and intensity of use

In addition to primary/secondary (clinical) outcomes, the presentation of process outcomes such as metrics of use and intensity of use (dose, exposure) and their operational definitions is critical. This does not only refer to metrics of attrition (13-b) (often a binary variable), but also to more continuous exposure metrics such as "average session length". These must be accompanied by a technical description how a metric like a "session" is defined (e.g., timeout after idle time) [1] (report under item 6a).

| | 1 | 2 | 3 | 4 | 5 | |
|---|---|---|---|---|---|---|
| subitem not at all important | ○ | ○ | ○ | ◉ | ○ | essential |

## Does your paper address subitem 17a-i?

Copy and paste relevant sections from the manuscript (include quotes in quotation marks "like this" to indicate direct quotes from your manuscript), or elaborate on this item by providing additional information not in the ms, or briefly explain why the item is not applicable/relevant for your study

We reported the completion rate. See Table 6.

## 17b) For binary outcomes, presentation of both absolute and relative effect sizes is recommended

## Does your paper address CONSORT subitem 17b? *

Copy and paste relevant sections from the manuscript (include quotes in quotation marks "like this" to indicate direct quotes from your manuscript), or elaborate on this item by providing additional information not in the ms, or briefly explain why the item is not applicable/relevant for your study

We used continuous outcome. This item is not applicable to the study.

## 18) Results of any other analyses performed, including subgroup analyses and adjusted analyses, distinguishing pre-specified from exploratory

### Does your paper address CONSORT subitem 18? *

Copy and paste relevant sections from the manuscript (include quotes in quotation marks "like this" to indicate direct quotes from your manuscript), or elaborate on this item by providing additional information not in the ms, or briefly explain why the item is not applicable/relevant for your study

The item is not applicable to the study. We did not perform any subgroup analyses.

### 18-i) Subgroup analysis of comparing only users

A subgroup analysis of comparing only users is not uncommon in ehealth trials, but if done, it must be stressed that this is a self-selected sample and no longer an unbiased sample from a randomized trial (see 16-iii).

| | 1 | 2 | 3 | 4 | 5 | |
|---|---|---|---|---|---|---|
| subitem not at all important | ○ | ○ | ○ | ◉ | ○ | essential |

### Does your paper address subitem 18-i?

Copy and paste relevant sections from the manuscript (include quotes in quotation marks "like this" to indicate direct quotes from your manuscript), or elaborate on this item by providing additional information not in the ms, or briefly explain why the item is not applicable/relevant for your study

The item is not applicable to the study. We did not perform any subgroup analyses.

### 19) All important harms or unintended effects in each group

(for specific guidance see CONSORT for harms)

### Does your paper address CONSORT subitem 19? *

Copy and paste relevant sections from the manuscript (include quotes in quotation marks "like this" to indicate direct quotes from your manuscript), or elaborate on this item by providing additional information not in the ms, or briefly explain why the item is not applicable/relevant for your study

We did not identify harms or unintended effect in the study.

### 19-i) Include privacy breaches, technical problems

Include privacy breaches, technical problems. This does not only include physical “harm” to participants, but also incidents such as perceived or real privacy breaches [1], technical problems, and other unexpected/unintended incidents. “Unintended effects” also includes unintended positive effects [2].

| | 1 | 2 | 3 | 4 | 5 | |
|---|---|---|---|---|---|---|
| subitem not at all important | ○ | ○ | ◉ | ○ | ○ | essential |

### Does your paper address subitem 19-i?

Copy and paste relevant sections from the manuscript (include quotes in quotation marks "like this" to indicate direct quotes from your manuscript), or elaborate on this item by providing additional information not in the ms, or briefly explain why the item is not applicable/relevant for your study

We did not identify harms or unintended effect in the study.

### 19-ii) Include qualitative feedback from participants or observations from staff/researchers

Include qualitative feedback from participants or observations from staff/researchers, if available, on strengths and shortcomings of the application, especially if they point to unintended/unexpected effects or uses. This includes (if available) reasons for why people did or did not use the application as intended by the developers.

| | 1 | 2 | 3 | 4 | 5 | |
|---|---|---|---|---|---|---|
| subitem not at all important | ○ | ○ | ◉ | ○ | ○ | essential |

### Does your paper address subitem 19-ii?

Copy and paste relevant sections from the manuscript (include quotes in quotation marks "like this" to indicate direct quotes from your manuscript), or elaborate on this item by providing additional information not in the ms, or briefly explain why the item is not applicable/relevant for your study

We did not identify harms or unintended effect in the study.

## DISCUSSION

## 22) Interpretation consistent with results, balancing benefits and harms, and considering other relevant evidence

NPT: In addition, take into account the choice of the comparator, lack of or partial blinding, and unequal expertise of care providers or centers in each group

### 22-i) Restate study questions and summarize the answers suggested by the data, starting with primary outcomes and process outcomes (use)

Restate study questions and summarize the answers suggested by the data, starting with primary outcomes and process outcomes (use).

| | 1 | 2 | 3 | 4 | 5 | |
|---|---|---|---|---|---|---|
| subitem not at all important | ○ | ○ | ○ | ◉ | ○ | essential |

### Does your paper address subitem 22-i? *

Copy and paste relevant sections from the manuscript (include quotes in quotation marks "like this" to indicate direct quotes from your manuscript), or elaborate on this item by providing additional information not in the ms, or briefly explain why the item is not applicable/relevant for your study

Like this, "This is one of the first studies to compare the validity and reliability of the videoconference and telephone functions of an mHealth app for collecting functional status data in stroke patients after rehabilitation hospitalization, and to examine the feasibility and acceptability of using these modes of administration for data collection. We examined these questions prospectively in a cohort of patients who were discharged from inpatient stroke rehabilitation by comparing videoconference and telephone follow-up assessments, as well as comparing these 2 modes of administration to the home visit assessment as the gold standard.
Our findings revealed that videoconference, but not telephone, administration was as valid and reliable as face-to-face, home visit assessment at both 2-week and 3-month follow-up periods. "

### 22-ii) Highlight unanswered new questions, suggest future research

Highlight unanswered new questions, suggest future research.

| | 1 | 2 | 3 | 4 | 5 | |
|---|---|---|---|---|---|---|
| subitem not at all important | ○ | ○ | ○ | ◉ | ○ | essential |

Does your paper address subitem 22-ii?

Copy and paste relevant sections from the manuscript (include quotes in quotation marks "like this" to indicate direct quotes from your manuscript), or elaborate on this item by providing additional information not in the ms, or briefly explain why the item is not applicable/relevant for your study

Like this, "Future research should explore mHealth assessments for measuring other health outcomes in post-stroke individuals after discharge from the hospital. Another limitation includes measuring acceptability through 3 self-constructed items (satisfaction, comfort, and confidence), which are limited in their measurement of this construct and do not provide actionable data to inform future research. Even though these self-constructed items allow for efficient quantification of acceptability, future research should add a qualitative or mixed methods approach to provide additional interpretation and meaning to the quantitative results [52]. Furthermore, this study did not record the amount of time required for performing videoconference and telephone follow-up assessments. Future research should measure the duration of these 2 follow-up assessments and compare them with the time needed for the home visit assessment to provide additional validation evidence. Future research is also needed to conduct a randomized controlled trial comparing these 2 modes of mobile administration to traditional telephone interview method (eg, landline phone service) for stroke patients as a control condition. "

## 20) Trial limitations, addressing sources of potential bias, imprecision, and, if relevant, multiplicity of analyses

20-i) Typical limitations in ehealth trials

Typical limitations in ehealth trials: Participants in ehealth trials are rarely blinded. Ehealth trials often look at a multiplicity of outcomes, increasing risk for a Type I error. Discuss biases due to non-use of the intervention/usability issues, biases through informed consent procedures, unexpected events.

| | 1 | 2 | 3 | 4 | 5 | |
|---|---|---|---|---|---|---|
| subitem not at all important | ○ | ○ | ○ | ● | ○ | essential |

### Does your paper address subitem 20-i? *

Copy and paste relevant sections from the manuscript (include quotes in quotation marks "like this" to indicate direct quotes from your manuscript), or elaborate on this item by providing additional information not in the ms, or briefly explain why the item is not applicable/relevant for your study

Like this "Our study has several limitations. First, patients were recruited from 1 rehabilitation hospital in a coastal province in eastern China; therefore, results may not generalize to persons living in other regions. Second, the current study only included participants who owned a smartphone and served as their own informant to complete the assessment; patients without a smartphone and those with severe cognitive or communication impairments may have been excluded from this study. Third, the MBI was the only outcome assessment used in the study. "

## 21) Generalisability (external validity, applicability) of the trial findings

NPT: External validity of the trial findings according to the intervention, comparators, patients, and care providers or centers involved in the trial

### 21-i) Generalizability to other populations

Generalizability to other populations: In particular, discuss generalizability to a general Internet population, outside of a RCT setting, and general patient population, including applicability of the study results for other organizations

| | 1 | 2 | 3 | 4 | 5 | |
|---|---|---|---|---|---|---|
| subitem not at all important | ○ | ○ | ○ | ● | ○ | essential |

### Does your paper address subitem 21-i?

Copy and paste relevant sections from the manuscript (include quotes in quotation marks "like this" to indicate direct quotes from your manuscript), or elaborate on this item by providing additional information not in the ms, or briefly explain why the item is not applicable/relevant for your study

Like this "First, patients were recruited from 1 rehabilitation hospital in a coastal province in eastern China; therefore, results may not generalize to persons living in other regions. Second, the current study only included participants who owned a smartphone and served as their own informant to complete the assessment; patients without a smartphone and those with severe cognitive or communication impairments may have been excluded from this study. "

### 21-ii) Discuss if there were elements in the RCT that would be different in a routine application setting

Discuss if there were elements in the RCT that would be different in a routine application setting (e.g., prompts/reminders, more human involvement, training sessions or other co-interventions) and what impact the omission of these elements could have on use, adoption, or outcomes if the intervention is applied outside of a RCT setting.

| | 1 | 2 | 3 | 4 | 5 | |
|---|---|---|---|---|---|---|
| subitem not at all important | ○ | ○ | ◉ | ○ | ○ | essential |

### Does your paper address subitem 21-ii?

Copy and paste relevant sections from the manuscript (include quotes in quotation marks "like this" to indicate direct quotes from your manuscript), or elaborate on this item by providing additional information not in the ms, or briefly explain why the item is not applicable/relevant for your study

We described the use of technology to monitor post-hospitalization functional status in stroke is uncommon but may be a potential viable approach to  understand patient's functional recovery. Like this, "Furthermore, inadequate manpower and financial concerns restrict the implementation of home visit assessments for all patients after hospitalization. Instead, other studies [40] have recommended home-based telemedicine as a viable option in the delivery of post-stroke recovery programs, because telemedicine has shown promising results in improving the overall health of stroke patients and in supporting caregivers while being delivered by therapists from a distance. The use of technologies appears to be a novel potential approach for the therapist's assessment of patient performance in home settings. Our findings concur with this notion that an app-based videoconference can be used to assess the functional performance of stroke patients in home settings. Videoconference may augment other technology-enabled solutions to provide a means of conducting future clinical trials aimed to evaluate the outcomes of any rehabilitation program

## OTHER INFORMATION

### 23) Registration number and name of trial registry

Does your paper address CONSORT subitem 23? *

Copy and paste relevant sections from the manuscript (include quotes in quotation marks "like this" to indicate direct quotes from your manuscript), or elaborate on this item by providing additional information not in the ms, or briefly explain why the item is not applicable/relevant for your study

Like this, "This trial was registered with the Chinese Clinical Trial Registry (ChiCTR): ChiCTR1900027626."

### 24) Where the full trial protocol can be accessed, if available

Does your paper address CONSORT subitem 24? *

Cite a Multimedia Appendix, other reference, or copy and paste relevant sections from the manuscript (include quotes in quotation marks "like this" to indicate direct quotes from your manuscript), or elaborate on this item by providing additional information not in the ms, or briefly explain why the item is not applicable/relevant for your study

We listed the information in the abstract. Like this: "Trial Registration: chictr.org.cn ChiCTR1900027626; http://www.chictr.org.cn/edit.aspx?pid=44831&htm=4"

### 25) Sources of funding and other support (such as supply of drugs), role of funders

Does your paper address CONSORT subitem 25? *

Copy and paste relevant sections from the manuscript (include quotes in quotation marks "like this" to indicate direct quotes from your manuscript), or elaborate on this item by providing additional information not in the ms, or briefly explain why the item is not applicable/relevant for your study

We listed it in the acknowledgements. Like this "The contents do not necessarily represent the policy of the funding agencies. We certify that all financial and material support for this research and work are clearly identified in the manuscript. We acknowledge Megen Devine at Washington University for her editorial assistance with this manuscript. This study was funded by the 12th Five-Year Plan Supporting Project of Ministry of Science and Technology of the People's Republic of China (2013BAI10B01). It was supported by Collaborative Innovation Center of Rehabilitation Technology. The US National Institutes of Health (K01HD095388) supported a portion of senior author (A.W.K.W.)'s effort. "

## X27) Conflicts of Interest (not a CONSORT item)

### X27-i) State the relation of the study team towards the system being evaluated

In addition to the usual declaration of interests (financial or otherwise), also state the relation of the study team towards the system being evaluated, i.e., state if the authors/evaluators are distinct from or identical with the developers/sponsors of the intervention.

| | 1 | 2 | 3 | 4 | 5 | |
|---|---|---|---|---|---|---|
| subitem not at all important | ○ | ○ | ○ | ◉ | ○ | essential |

### Does your paper address subitem X27-i?

Copy and paste relevant sections from the manuscript (include quotes in quotation marks "like this" to indicate direct quotes from your manuscript), or elaborate on this item by providing additional information not in the ms, or briefly explain why the item is not applicable/relevant for your study

Like this "Conflict of Interest: All authors declare that they have no conflicts of [illegible]

## About the CONSORT EHEALTH checklist

### As a result of using this checklist, did you make changes in your manuscript? *

- ○ yes, major changes
- ◉ yes, minor changes
- ○ no

### What were the most important changes you made as a result of using this checklist?

I added more detailed information about the mHealth components.

How much time did you spend on going through the checklist INCLUDING making changes in your manuscript *

2 hours.

As a result of using this checklist, do you think your manuscript has improved? *

- (x) yes
- ( ) no
- ( ) 其他:

Would you like to become involved in the CONSORT EHEALTH group?

This would involve for example becoming involved in participating in a workshop and writing an "Explanation and Elaboration" document

- ( ) yes
- (x) no
- ( ) 其他:

Any other comments or questions on CONSORT EHEALTH

您的回答

## STOP - Save this form as PDF before you click submit

To generate a record that you filled in this form, we recommend to generate a PDF of this page (on a Mac, simply select "print" and then select "print as PDF") before you submit it.

When you submit your (revised) paper to JMIR, please upload the PDF as supplementary file.

Don't worry if some text in the textboxes is cut off, as we still have the complete information in our database. Thank you!

## Final step: Click submit !

Click submit so we have your answers in our database!

提交

切勿通过 Google 表单提交密码。

此内容不是由 Google 所创建，Google 不对其作任何担保。 举报滥用行为 - 服务条款 - 隐私权政策

Google 表单
